# Supplementary material for: SARS-CoV-2 tests, confirmed infections and COVID-19-related hospital admissions in children and young people: birth cohort study
Source: BMJ Paediatr Open. 2022 Aug 29;6(1):e001545. doi: 10.1136/bmjpo-2022-001545 (PMC9437731; doi:10.1136/bmjpo-2022-001545)
Supplement: Supplementary data [file bmjpo-2022-001545supp001.pdf]

SARS-CoV-2 tests, confirmed infections and hospital admissions in children and young people: birth cohort study

Supplementary material

Supplementary text 1

Outcome definitions

We included all SARS-CoV-2 PCR tests recorded between1st August 2020 and 31st December 2020 in the COVID19 Tests Dataset. The samples were collected in hospitals, primary care, via national testing centres, or self-collection via home test kits. We did not include antigen (lateral flow device) test results, as only 5% of test results in the cohort during the study period were from antigen tests. We defined as duplicate tests multiple tests taken on the same day, in the same CYP, with the same result, irrespective of whether they were taken at different locations. All duplicate tests, whether positive or negative, were excluded when calculating testing rates. A PCR confirmed infection was defined as the first record of a positive SARS-CoV-2 PCR test result (the index positive test) recorded in the COVID19 Tests dataset between1st August 2020 and 31st December 2020. Public Health Scotland recommends excluding all repeat positive tests within 90 days of the index positive sample date, and less than 5 CYP had multiple positive results beyond this time period. Therefore, only the first positive SARS-CoV-2 PCR test result for each child was included when calculating rates of PCR-confirmed SARS-CoV-2 infections.

We included all COVID-19-related hospital admissions between 1st February and 31st December 2020. To define COVID-19 related hospital admissions, we first linked episodes in the hospital admission dataset (Scottish Morbidity Record-01; Table 1) into admissions by assuming that episodes where the difference between the admission date and previous discharge date was ≤1 day[22] indicated the same admission. Second, we identified COVID-19 related admissions where: (i) an individual had tested positive for SARS-CoV-2 up to 14 days prior to hospital admission, on the day of admission, or in between the hospital admission and discharge date, and/or (ii) an International Classification of Diseases-version 10 (ICD-10) diagnostic code for COVID-19 (U07.1 – U07.2) had been recorded during an admission as a primary or secondary diagnosis.

Hardelid P, *et al. BMJ Paediatrics Open* 2022; 6:e001545. doi: 10.1136/bmjpo-2022-001545

Since the ICD-10 code for PIMS-TS (U07.5) was introduced at the end of the follow-up period, we used other ICD-10 codes indicating systemic inflammatory response syndrome of infectious origin without organ failure (R65X), cardiogenic shock (R57X) or other specified systemic involvement of connective tissue (M35.8), suggestive of PIMS-TS recorded during an admission which had a positive SARS-CoV-2 PCR test within 28 day prior to the admission date.

A COVID-19-related intensive care unit (ICU) stay was defined where a child had an SMR-01 episode with 'significant facility' recorded with a positive SARS-CoV-2 PCR test to 21 days prior to the start of, or during, the ICU stay. ICU episodes where the difference between the ICU admission date and previous ICU discharge date was ≤1 day were assumed to indicate the same ICU stay.

Supplementary text 2

Risk factors for testing

Testing rates varied by age group and chronic conditions; it was higher in children aged 1-4 years, young adults (age 18-22 years), and those with more than one chronic condition (Supplementary Table 2 & 3). Among children aged <5 years old, testing rates were higher in children from a higher socio-economic position, whereas among CYP aged 12-22 years, testing rates were higher in lower socio-economic groups.

The all-age model suggested increasing age and chronic conditions were strongly associated with being tested (Supplementary Table 4). In age-group stratified analyses, a history of chronic conditions was strongly associated with higher testing rates (Supplementary Table 5), particularly among infants.

Hardelid P, *et al. BMJ Paediatrics Open* 2022; 6:e001545. doi: 10.1136/bmjpo-2022-001545

Supplementary Tables

Supplementary TABLE 1: Cohort baseline characteristics (*n*=1,226,855)

	Number	%
Sex		
Male	628,410	51.2
Female	598,445	48.8
missing	*0*	*0*
Age (years)*		
Median 10.8 years (IQR 5-17) y		
<1 year	92,539	7.5
1-4 years	206,677	16.9
5-11 years	326,455	26.6
12-17 years	358,195	29.2
18-22 years	242,989	19.8
Missing	*0*	*0*
Socio economic position**		
High	136,938	11.2
Middle	582,342	47.5
Low	507,563	41.4
Missing	*12*	*0*
Chronic conditions***		
None	1,128,971	92.0
One	78,016	6.4
More than one type	19,868	1.6
Gestational age (weeks) (aged* <5yr, *n*=292,289)		
Pre-term (<37 weeks)	23,825	8.0
Normal/Post-term (≥37 weeks)	268,464	89.7
Missing	*6,927*	*2.3*
Number of older siblings (aged*<5yr, *n*=289,800)		
None	124,289	41.5
One	102,944	34.4
Two or more	62,567	20.9
Missing	*9,416*	3.2
BMI *** (aged* 5-17, *n*=550,874)		
Underweight	8,930	1.30
Normal	421,182	60.2
Overweight/Obese	120,762	17.6
missing	*142,776*	*20.9*

* As on 1st February 2020; aged<1yr includes those born between 1 Feburary2020 and 31 December 2020. ** From UK National Statistics Socio-economic Classification (NS-SEC): SEP (managerial and professional occupations), middle SEP (intermediate occupations), low SEP (routine and manual occupations). ***Includes any chronic conditions recorded in the hospital records in the previous five years. ***As recorded in the Child Health Surveillance Programme-School at aged 5 and standardised according to the British 1990 growth reference standards (Cole 1998): underweight (<5th percentile), normal weight (5th to <85th percentile), overweight/obese (≥85th percentile).

Supplementary Table 2 Rate testing by age group (age 0-4 years) per 1,000 CYP-years

	**Age <1 year**				**Age 1-4 years**			
	Events	Rate	95%LCI	95%UCI	Events	Rate	95%LCI	95%UCI
Overall	9509	482	473	492	59176	702	696	708
SEX								
Male	5256	519	506	534	32284	743	735	752
Female	4253	443	430	457	26892	658	650	666
Socio-economic position								
High	1368	551	522	581	9417	937	918	956
Middle	4417	466	452	480	29119	729	720	737
Low	3724	481	466	497	20637	602	594	610
CHRONIC CONDITIONS								
None	7678	408	399	417	50790	657	651	663
One	980	1323	1242	1408	5712	997	971	1023
More than one	851	5481	5124	5861	2674	2082	2005	2163
GESTATIONAL AGE								
pre-term	8099	457	448	468	52447	693	687	699
Term/post-term	1207	797	753	843	5566	818	796	839
NUMBER OLDER SIBLINGS								
None	4030	479	464	494	26920	770	761	779
One	3182	489	472	506	20177	692	682	701
More than one	1996	487	466	509	10371	589	577	600
BMI								
underweight	-	-	-	-	-	-	-	-
normal	-	-	-	-	-	-	-	-
overweight/obese	-	-	-	-	-	-	-	-

Supplementary Table 3 Rate of testing by age group (age 5-22 years) per 1,000 CYP-years

	**Age 5-11 years**				**Age 12-17 years**				**Age 18-22 years**			
	Events	Rate	95%LCI	95%UCI	Events	Rate	95%LCI	95%UCI	Events	Rate	95%LCI	95%UCI
Overall	92007	583	580	587	79771	623	619	627	137939	1364	1357	1371
SEX												
Male	49757	622	616	628	45322	586	581	592	46290	895	887	903
Female	42250	558	553	564	46831	635	630	641	91649	1854	1842	1866
Socio-economic position												
High	10528	591	579	603	10059	531	521	541	9961	1198	1175	1222
Middle	41274	593	587	600	39678	612	606	618	78068	1390	1380	1399
Low	40203	588	582	595	42416	631	625	637	49909	1362	1350	1374
CHRONIC CONDITIONS												
None	80747	552	549	556	70707	592	587	596	117695	1303	1296	1311
One	8000	850	832	869	6395	944	921	967	14918	1789	1761	1818
More than one	3260	1538	1486	1592	2669	1530	1473	1590	5326	2137	2080	2195
GESTATIONAL AGE												
pre-term	-	-	-	-	-	-	-	-	-	-	-	-
term	-	-	-	-	-	-	-	-	-	-	-	-
post-term	-	-	-	-	-	-	-	-	-	-	-	-
NUMBER OLDER SIBLINGS												
None	-	-	-	-	-	-	-	-	-	-	-	-
One	-	-	-	-	-	-	-	-	-	-	-	-
More than one	-	-	-	-	-	-	-	-	-	-	-	-
BMI												
underweight	1009	626	589	666	1250	644	610	681	-	-	-	-
normal	49281	567	562	572	46792	594	589	600	-	-	-	-
overweight/obese	15287	588	579	598	14415	639	628	649	-	-	-	-

Hardelid P, *et al. BMJ Paediatrics Open* 2022; 6:e001545. doi: 10.1136/bmjpo-2022-001545

Supplementary Table 4 Results of models adjusted for age group, sex, socio-economic position, and history of chronic conditions

	Testing*		PCR confirmed infection**		Admission***	
	Adj IRR	95%CI	Adj HR	95%CI	Adj HR	95%CI
AGE GROUP						
<1year	0.94	0.92, 0.95	0.76	0.70, 0.84	10.11	7.14, 14.32
1-4 years	1.14	1.12, 1.15	0.58	0.54, 0.62	1.11	0.74, 1.68
5-11 years	1.00	-	1	-	1	-
12-17 years	1.15	1.13, 1.16	2.38	2.28, 2.48	1.25	0.87, 1.80
18-22 years	1.89	1.87, 1.91	3.13	3.00, 3.26	2.32	1.66, 3.23
SEX						
Male	1	-	1	-	1	-
Female	1.16	1.15, 1.17	1.05	1.02, 1.08	1.11	0.89, 1.39
Socio-economic position						
High	1	-	1	-	1	-
Middle	1.00	0.98, 1.01	0.95	0.91, 1.00	1.34	0.86, 2.11
Low	0.96	0.95, 0.98	0.85	0.81 0.89	1.53	0.98. 2.40
CHRONIC CONDITIONS						
None	1	-	1	-	1	-
One	1.38	1.36, 1.40	0.75	0.71, 0.79	7.55	5.79, 9.86
More than one	1.85	1.81, 1.88	0.61	0.55, 0.68	26.17	19.79, 34.59

PCR, Polymerase Chain Reaction; Adj IRR, Adjusted Incidence Risk Ratio; Adj HR, Adjusted Hazard Ratio; CI, Confidence Intervals.

Footnotes:

* Wald test for interaction: age and sex p<0.0001; age and socio-economic position p<0.0001; age and chronic conditions p<0.0001;

**interaction: age and sex p<0.0001 , age and socio-economic position p<0.0001 , age and chronic conditions p=0.0004; global test to check proportionality assumption p<0.0001 (for age, socio-economic position, chronic conditions).

*** interaction: age and sex p= 0.045 , age and NS-SEC p= 0.94 , age and chronic conditions p =0.26; global test to check proportionality assumption p 0.0009 (for age, chronic condition

Supplementary Table 5 Incidence Risk Ratio of being tested by age group mutually adjusted for sex, socio-economic status, history of chronic conditions and parity and pre-term (age<5 years) and BMI (aged 5-17 years)

	**Age <1 year**			**Age 1-4 years**			**Age 5-11 years**			**Age 12-17 years**		
N CYP in model	89202			200590			310670			231202		
N tests in model	94799			213258			323020			247143		
	IRR	95%LCI	95%UCI	IRR	95%LCI	95%UCI	IRR	95%LCI	95%UCI	IRR	95%LCI	95%UCI
SEX												
Male	1.00	-	-	1.00	-	-	1.00	-	-	1.00	-	-
Female	0.89	0.86	0.92	0.91	0.90	0.93	0.91	0.89	0.92	1.15	1.13	1.17
SOCIO-ECONOMIC POSITION												
High	1.00	-	-	1.00	-	-	1.00	-	-	1.00	-	-
Middle	0.76	0.73	0.80	0.84	0.82	0.86	1.02	1.00	1.05	1.09	1.06	1.13
Low	0.62	0.60	0.65	0.76	0.74	0.78	1.01	0.98	1.03	1.10	1.06	1.13
CHRONIC CONDITIONS												
None	1.00	-	-	1.00	-	-	1.00	-	-	1.00	-	-
One	2.13	2.02	2.25	1.44	1.40	1.48	1.46	1.42	1.49	1.48	1.43	1.53
More than one	3.89	3.66	4.14	2.22	2.11	2.33	2.14	2.03	2.25	1.91	1.80	2.02
GESTATIONAL AGE												
Pre-term	1.10	1.04	1.15	1.07	1.04	1.10	-	-	-	-	-	-
Term/post-term	1.00	-	-	1.00	-	-	-	-	-	-	-	-
NUMBER OLDER SIBLINGS												
None	1.00	-	-	1.00	-	-	-	-	-	-	-	-
One	0.99	0.95	1.02	0.91	0.89	0.93	-	-	-	-	-	-
More than one	0.86	0.82	0.90	0.82	0.80	0.84	-	-	-	-	-	-
BMI												
underweight	-	-	-	-	-	-	1.04	0.98	1.11	1.10	1.02	1.19
normal	-	-	-	-	-	-	1.00	-	-	1.00	-	-
overweight/obese	-	-	-	-	-	-	1.03	1.01	1.04	1.05	1.03	1.08

Hardelid P, *et al. BMJ Paediatrics Open* 2022; 6:e001545. doi: 10.1136/bmjpo-2022-001545

Supplementary Table 6 Rate of PCR confirmed infections by age group per 1,000 CYP-years – extra variables

	**Age<1 year**				**Age 1-4 years**			
	Events	Rate	95%LCI	95%UCI	Events	Rate	95%LCI	95%UCI
	223	80	70	91	1136	62	58	65
GESTATIONAL AGE								
pre-term	192	78	68	90	1022	62	59	66
term/post-term	24	85	57	126	89	53	43	66
NUMBER OLDER SIBLINGS								
None	109	92	76	110	541	65	60	71
One	58	60	46	77	358	57	51	63
More than one	48	86	65	114	201	61	53	70
	**Age 5-11 years**				**Age 12-17 years**			
	Events	Rate	95%LCI	95%UCI	Events	Rate	95%LCI	95%UCI
	3039	96	93	100	4929	193	188	198
BMI								
underweight	38	112	82	154	72	179	142	225
normal	1723	101	96	106	2791	182	175	189
overweight/obese	545	104	96	113	918	198	186	211

Hardelid P, *et al. BMJ Paediatrics Open* 2022; 6:e001545. doi: 10.1136/bmjpo-2022-001545

Supplementary Table 7 Time to PCR confirmed infection: hazard ratios (HR) by age group mutually adjusted for sex, socio-economic status, history of chronic conditions and parity and pre-term (age<5 years) and BMI (age 5-17 years)

	**Age <1 years**			**Age 1-4 years**			**Age 5-11 years**			**Age 12-17 years**		
N CYP in model	9396			47930			46753			61991		
N events in model												
	HR	95%LCI	95%UCI	HR	95%LCI	95%UCI	HR	95%LCI	95%UCI	HR	95%LCI	95%UCI
SEX												
Male	1.00	-	-	1.00	-	-	1.00	-	-	1.00	-	-
Female	1.14	0.92	1.40	1.09	0.97	1.21	1.09	1.00	1.19	1.21	1.14	1.28
SOCIO-ECONOMIC Position												
High	1.00	-	-	1.00	-	-	1.00	-	-	1.00	-	-
Middle	1.43	1.00	2.04	1.30	1.08	1.55	1.24	1.06	1.46	1.07	0.97	1.17
Low	1.46	1.01	2.10	1.34	1.11	1.61	1.15	0.98	1.34	0.95	0.87	1.05
CHRONIC CONDITIONS												
None	1.00	-	-	1.00	-	-	1.00	-	-	1.00	-	-
One	0.64	0.40	1.01	0.76	0.62	0.94	0.86	0.72	1.02	0.80	0.71	0.90
More than one	1.29	0.73	2.27	0.82	0.57	1.19	0.85	0.60	1.20	0.58	0.44	0.75
GESTATIONAL AGE												
pre-term	0.82	0.56	1.20	0.84	0.68	1.04	-	-	-	-	-	-
Term/post-erm	1.00	-	-	1.00	-	-	-	-	-	-	-	-
NUMBER OLDER SIBLINGS												
None	1.00	-	-	1.00	-	-	-	-	-	-	-	-
One	0.63	0.49	0.80	0.86	0.76	0.98	-	-	-	-	-	-
More than one	0.80	0.61	1.06	0.91	0.78	1.06	-	-	-	-	-	-
BMI												
underweight	-	-	-	-	-	-	1.03	0.72	1.48	1.04	0.84	1.28
normal	-	-	-	-	-	-	1.00	-	-	1.00	-	-
overweight/obese	-	-	-	-	-	-	1.06	0.96	1.18	1.08	1.01	1.15

Supplementary Table 8 Rate of COVID-related admissions by age group per 100,000 CYP-years – extra variables

	**Age<1 years**				**Age 1-4 years**			
	Events	Rate	95%LCI	95%UCI	Events	Rate	95%LCI	95%UCI
	53	121	92	158	51	27	21	36
GESTATIONAL AGE								
pre-term	42	106	79	144	41	24	18	33
term/post-term	10	290	156	538	8	53	27	106
NUMBER OLDER SIBLINGS								
None	24	129	86	192	*	26	17	40
One	16	109	67	178	*	25	15	40
More than one	11	120	67	217	*	31	17	54
	**Age 5-11 years**				**Age 12-17 years**			
	Events	Rate	95%LCI	95%UCI	Events	Rate	95%LCI	95%UCI
	55	16	12	21	49	18	13	23
BMI								
underweight	*	54	13	215	*	24	*	168
normal	*	13	9	19	*	12	*	18
overweight/obese	*	15	8	29	*	25	14	44

*Redacted due to small numbers in some groups

Supplementary Table 9 Time-to-COVID related admission: hazard ratios (HR) by age group mutually adjusted for sex, socio-economic status, history of chronic conditions and parity and pre-term (age<5 years) and BMI (aged 5-17 years)

	**Age <1 year**			**Age 1-4 years**			**Age 5-11 years**			**Age 12-17 years**		
N CYP in model	89197			244438			235396			316309		
N events in model												
	HR	95%LCI	95%UCI	HR	95%LCI	95%UCI	HR	95%LCI	95%UCI	HR	95%LCI	95%UCI
SEX												
Male	1.00	-	-	1.00	-	-	1.00	-	-	1.00	-	-
Female	0.81	0.50	1.31	1.58	0.92	2.71	0.71	0.34	1.50	0.87	0.50	1.52
SOCIO-ECONOMIC Position												
High	1.00	-	-	1.00	-	-	1.00	-	-	1.00	-	-
Middle	1.98	0.70	5.61	1.26	0.43	3.68	0.89	0.30	2.67	3.34	0.79	14.08
Low	2.46	0.87	6.95	1.91	0.67	5.45	0.52	0.16	1.67	2.49	0.59	10.62
CHRONIC CONDITIONS												
None	1.00	-	-	1.00	-	-	1.00	-	-	1.00	-	-
One	3.05	1.44	6.49	2.69	1.19	6.08	14.71	6.47	33.41	12.50	6.75	23.18
More than one	9.75	4.02	23.66	21.71	11.00	42.85	51.43	20.08	131.73	26.14	11.76	58.12
GESTATIONAL AGE												
pre-term	1.67	0.87	3.24	0.97	0.44	2.14	-	-	-	-	-	-
term	1.00	-	-	1.00	-	-	-	-	-	-	-	-
NUMBER OLDER SIBLINGS												
None	1.00	-	-	1.00	-	-	-	-	-	-	-	-
One	0.82	0.47	1.44	0.82	0.44	1.52	-	-	-	-	-	-
More than one	0.96	0.52	1.75	0.83	0.42	1.67	-	-	-	-	-	-
BMI												
underweight	-	-	-	-	-	-	1.75	0.23	13.05	2.22	0.53	9.27
normal	-	-	-	-	-	-	1.00	-	-	1.00	-	-
overweight/obese	-	-	-	-	-	-	0.94	0.40	2.21	1.41	0.76	2.60

Supplementary Table 10 Rate of 'specific' admissions (age 0-4 years) per 100,000 CYP-years

	**Age<1 years**				**Age 1-4 years**			
	Events	Rate	95%LCI	95%UCI	Events	Rate	95%LCI	95%UCI
	47	107.0	80.4	142.4	25	13.4	9.0	19.8
SEX								
Male	26	115.4	78.6	169.5	10	10.4	5.6	19.3
Female	21	98.1	64.0	150.5	15	16.6	10.0	27.5
Socio-economic position								
High	*	72.8	27.3	193.9	*	9.0	2.3	36.0
Middle	*	104.0	68.5	157.9	*	12.5	6.9	22.5
Low	*	121.5	79.2	186.3	*	15.7	8.9	27.7
CHRONIC CONDITIONS								
None	*	98.2	72.3	133.4	*	11.7	7.6	18.1
One	*	221.8	83.3	591.0	*	23.1	7.4	71.5
More than one	*	516.0	129.0	2063.0	*	68.0	17.0	271.9
GESTATIONAL AGE								
pre-term	38	96.2	70.0	132.2	*	13.1	8.6	19.9
term/post-term	8	231.7	115.9	463.3	*	20.0	6.4	61.9
NUMBER OLDER SIBLINGS								
None	21	112.6	73.4	172.6	*	14.2	7.9	25.7
One	15	102.2	61.6	169.5	*	13.9	7.2	26.7
More than one	9	98.4	51.2	189.1	*	12.8	5.3	30.7
BMI								
underweight	-	-	-	-	-	-	-	-
normal	-	-	-	-	-	-	-	-
overweight/obese	-	-	-	-	-	-	-	-

*redacted due to small numbers in some groups

Hardelid P, *et al. BMJ Paediatrics Open* 2022; 6:e001545. doi: 10.1136/bmjpo-2022-001545

Supplementary Table 11 Rate of 'specific' admissions (age 5-22 years) per 100,000 CYP-years

	**Age 5-11 years**				**Age 12-17 years**				**Age 18-22 years**			
	Events	Rate	95%LCI	95%UCI	Events	Rate	95%LCI	95%UCI	Events	Rate	95%LCI	95%UCI
	28	8.0	5.6	11.7	26	9.3	6.3	13.6	62	27.7	21.6	35.5
SEX												
Male	15	8.4	5.1	14.0	14	9.8	5.8	16.5	27	23.6	16.2	34.4
Female	13	7.7	4.4	13.2	12	8.8	5.0	15.5	35	31.9	22.9	44.4
Socio-economic position												
High	*	4.9	1.2	19.6	*	2.9	0.4	20.3	*	16.9	5.4	52.3
Middle	*	8.4	4.9	14.5	*	10.0	5.7	17.6	*	22.1	15.2	32.0
Low	*	8.5	5.0	14.7	*	10.4	6.0	17.9	*	39.0	27.4	55.5
CHRONIC CONDITIONS												
None	12	3.7	2.1	6.5	10	3.8	2.1	7.1	21	10.5	6.9	16.1
One	6	29.3	13.2	65.3	8	53.0	26.5	106.0	23	122.6	81.5	184.5
More than one	10	217.1	116.8	403.6	8	204.6	102.3	409.1	18	317.9	200.3	504.5
GESTATIONAL AGE												
pre-term	-	-	-	-	-	-	-	-	-	-	-	-
term	-	-	-	-	-	-	-	-	-	-	-	-
post-term	-	-	-	-	-	-	-	-	-	-	-	-
NUMBER OLDER SIBLINGS												
None	-	-	-	-	-	-	-	-	-	-	-	-
One	-	-	-	-	-	-	-	-	-	-	-	-
More than one	-	-	-	-	-	-	-	-	-	-	-	-
BMI												
underweight	*	53.9	13.5	215.4	*	0.0			-	-	-	-
normal	*	6.5	3.8	11.2	*	5.9	3.2	11.0	-	-	-	-
overweight/obese	*	10.0	4.5	22.3	*	12.4	5.6	27.7	-	-	-	-

*redacted due to small numbers in some groups

Hardelid P, *et al. BMJ Paediatrics Open* 2022; 6:e001545. doi: 10.1136/bmjpo-2022-001545

Supplementary Table 12 Time-to-COVID related admission (specific definition): hazard ratios (HR) by age group mutually adjusted for sex, socio-economic status and history of chronic conditions (age 0-4)

	**Age <1 year**			**Age 1-4 years**		
N CYP in model	92530			251884		
N events in model	47			25		
	HR	95%LCI	95%UCI	HR	95%LCI	95%UCI
SEX						
Male	1.00	-	-	1.00	-	-
Female	0.77	0.46	1.31	1.89	0.87	4.14
SOCIO-ECONOMIC Position						
High	1.00	-	-	1.00	-	-
Middle	1.40	0.54	3.63	1.62	0.37	7.17
Low	1.54	0.59	4.03	1.69	0.38	7.57
CHRONIC CONDITIONS						
None	1.00	-	-	1.00	-	-
One	2.28	0.91	5.73	2.52	0.87	7.36
More than one	4.28	1.04	17.59	5.54	1.30	23.70

Supplementary Table 13 Time-to-COVID related admission (specific definition): hazard ratios (HR) by age group mutually adjusted for sex, socio-economic status and history of chronic conditions (age 5-22)

	**Age 5-11 years**			**Age 12-17 years**			**Age 18-22 years**		
N CYP in model	347542			385664			268467		
N events in model	28			26			62		
	HR	95%LCI	95%UCI	HR	95%LCI	95%UCI	HR	95%LCI	95%UCI
SEX									
Male	1.00	-	-	1.00	-	-	1.00	-	-
Female	1.26	0.56	2.82	0.98	0.51	1.86	1.07	0.64	1.79
SOCIO-ECONOMIC Position									
High	1.00	-	-	1.00	-	-	1.00	-	
Middle	1.36	0.30	6.07	4.58	0.61	34.46	1.02	0.31	3.37
Low	1.02	0.22	4.66	4.34	0.58	32.47	1.67	0.51	5.49
CHRONIC CONDITIONS									
None	1.00	-	-	1.00	-	-	1.00	-	
One	7.87	2.68	23.07	11.14	5.05	24.58	9.89	5.41	18.08
More than one	64.09	25.87	158.82	48.19	22.33	104.01	28.22	15.06	52.89

Supplementary Figure 1 Flow chart describing creation of the final cohort

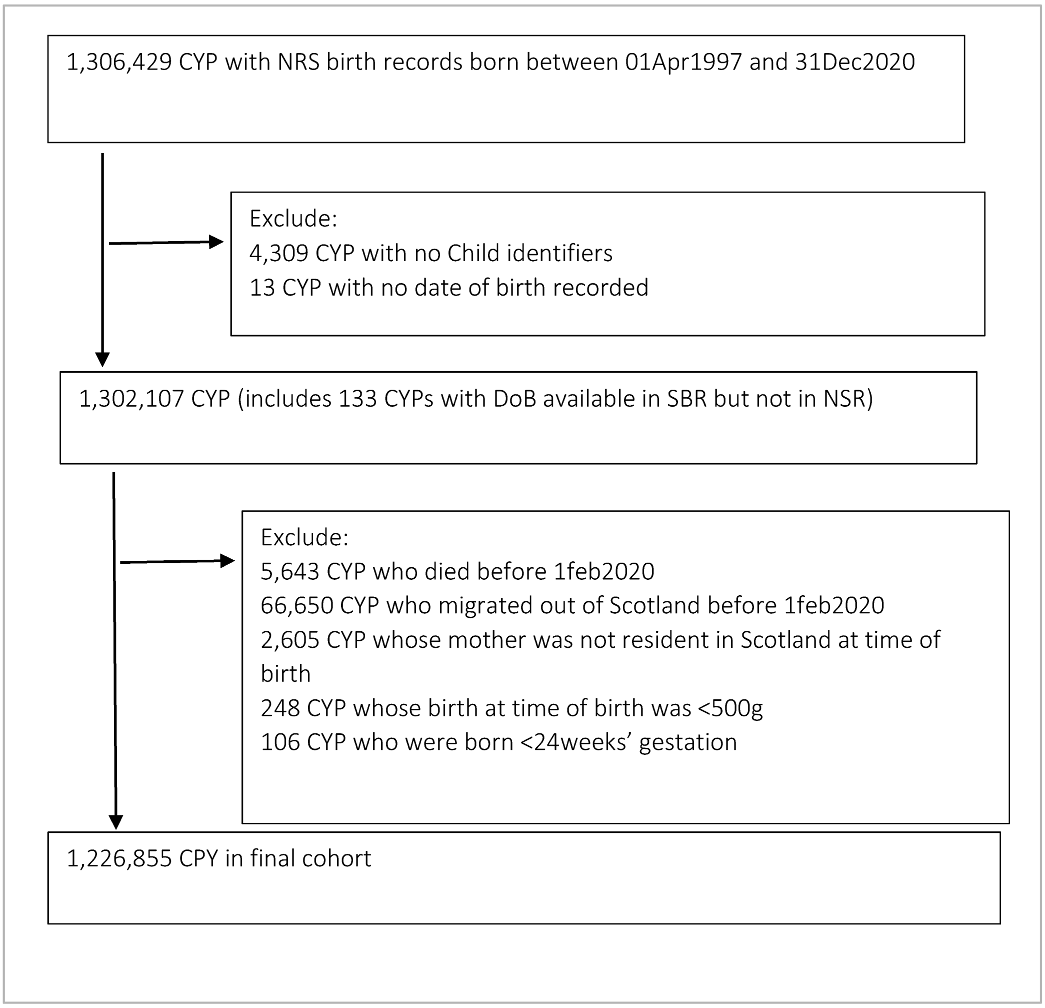

Supplementary Figure 2 Number of tests (bars) and positive tests (red) by age group and week of 2020 (note that the scale of the y-axis is not the same for all graphs)

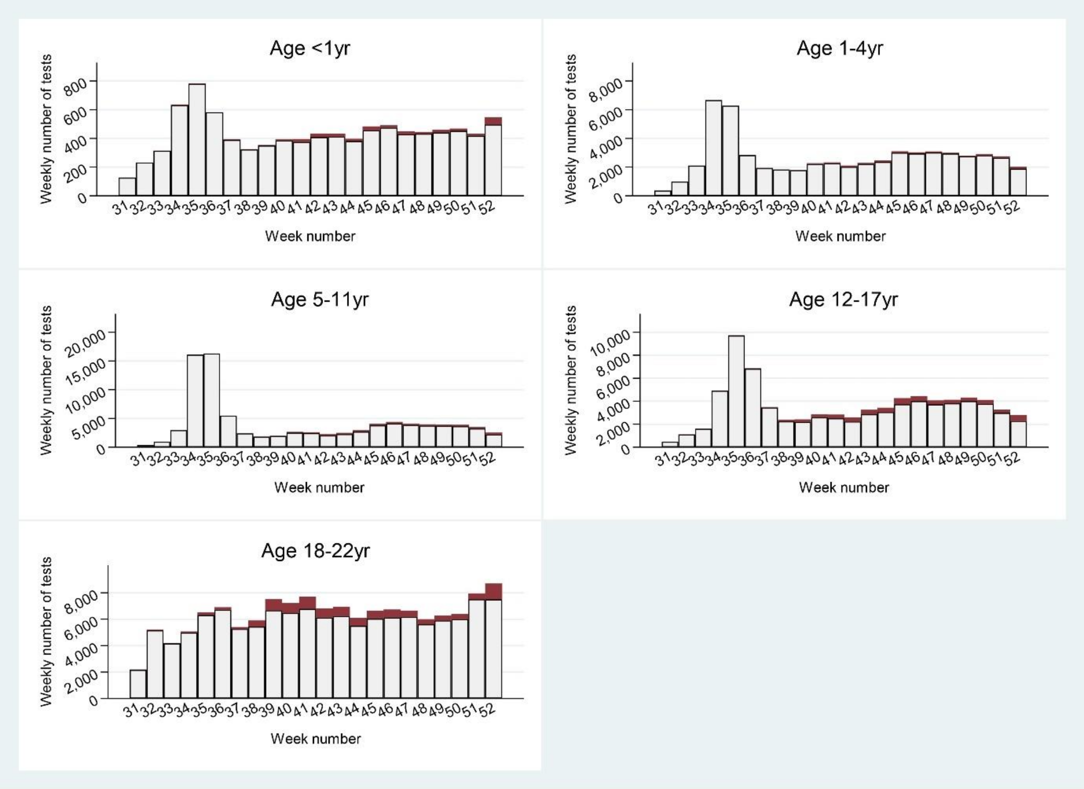

Hardelid P, *et al. BMJ Paediatrics Open* 2022; 6:e001545. doi: 10.1136/bmjpo-2022-001545
