# Supplementary material for: SARS-CoV-2 tests, confirmed infections and COVID-19-related hospital admissions in children and young people: birth cohort study
Source: BMJ Paediatr Open. 2022 Aug 29;6(1):e001545. doi: 10.1136/bmjpo-2022-001545 (PMC9437731; doi:10.1136/bmjpo-2022-001545)
Supplement: Author's manuscript [file bmjpo-2022-001545.draft_revisions.pdf]

BMJ Paediatrics Open

BMJ Paediatrics Open is committed to open peer review. As part of this commitment we make the peer review history of every article we publish publicly available.

When an article is published we post the peer reviewers' comments and the authors' responses online. We also post the versions of the paper that were used during peer review. These are the versions that the peer review comments apply to.

The versions of the paper that follow are the versions that were submitted during the peer review process. They are not the versions of record or the final published versions. They should not be cited or distributed as the published version of this manuscript.

BMJ Paediatrics Open is an open access journal and the full, final, typeset and author-corrected version of record of the manuscript is available on our site with no access controls, subscription charges or pay-per-view fees (http://bmjpaedsopen.bmj.com).

If you have any questions on BMJ Paediatrics Open's open peer review process please email info.bmjpo@bmj.com

BMJ Paediatrics Open

SARS-CoV-2 tests, confirmed infections and COVID-19 related hospital admissions in children and young people: birth cohort study

Journal:	*BMJ Paediatrics Open*
Manuscript ID	bmjpo-2022-001545
Article Type:	Original research
Date Submitted by the Author:	13-May-2022
Complete List of Authors:	Hardelid, Pia; University College London Great Ormond Street Institute of Child Health, Population, Policy & Practice Research and Teaching Department<br>Favarato, Graziella; University College London Great Ormond Street Institute of Child Health, Population, Policy & Practice Research and Teaching Department<br>Wijlaars, Linda; University College London Great Ormond Street Institute of Child Health, Population, Policy & Practice Research and Teaching Department<br>Fenton, Lynda; Public Health Scotland, Clinical and Public Health Intelligence Team<br>McMenamin, Jim; Public Health Scotland, Respiratory Infection Team<br>Clemens, Tom; The University of Edinburgh, School of Geosciences<br>Dibben, Chris; The University of Edinburgh, School of Geosciences<br>Milojevic, Ai; London School of Hygiene & Tropical Medicine, Department of Public Health, Environments and Society<br>Macfarlane, Alison; City University of London<br>Taylor, Jonathon; Tampere University, Faculty of Built Environment<br>Cunningham, Steven; University of Edinburgh, Centre for Inflammation Research<br>Wood, Rachael; University of Edinburgh
Keywords:	COVID-19, Epidemiology

SCHOLARONE™
Manuscripts

I, the Submitting Author has the right to grant and does grant on behalf of all authors of the Work (as defined in the below author licence), an exclusive licence and/or a non-exclusive licence for contributions from authors who are: i) UK Crown employees; ii) where BMJ has agreed a CC-BY licence shall apply, and/or iii) in accordance with the terms applicable for US Federal Government officers or employees acting as part of their official duties; on a worldwide, perpetual, irrevocable, royalty-free basis to BMJ Publishing Group Ltd ("BMJ") its licensees and where the relevant Journal is co-owned by BMJ to the co-owners of the Journal, to publish the Work in this journal and any other BMJ products and to exploit all rights, as set out in our licence.

The Submitting Author accepts and understands that any supply made under these terms is made by BMJ to the Submitting Author unless you are acting as an employee on behalf of your employer or a postgraduate student of an affiliated institution which is paying any applicable article publishing charge ("APC") for Open Access articles. Where the Submitting Author wishes to make the Work available on an Open Access basis (and intends to pay the relevant APC), the terms of reuse of such Open Access shall be governed by a Creative Commons licence – details of these licences and which Creative Commons licence will apply to this Work are set out in our licence referred to above.

Other than as permitted in any relevant BMJ Author's Self Archiving Policies, I confirm this Work has not been accepted for publication elsewhere, is not being considered for publication elsewhere and does not duplicate material already published. I confirm all authors consent to publication of this Work and authorise the granting of this licence.

Confidential: For Review Only

SARS-CoV-2 tests, confirmed infections and COVID-19 related hospital admissions in children and young people: birth cohort study

Pia Hardelid,[1†] Graziella Favarato,[1] Linda Wijlaars,[1] Lynda Fenton,[2] Jim McMenamin,[3] Tom Clemens,[4] Chris Dibben, [4] Ai Milojevic,[5] Alison Macfarlane,[6] Jonathon Taylor,[7] Steven Cunningham,[8*] Rachael Wood.[9*]

[1]Population, Policy and Practice Research and Teaching Department, University College London Great Ormond Street Institute of Child Health, London, UK

[2]Clinical and Public Health Intelligence Team, Public Health Scotland, Edinburgh, UK

[3] Respiratory Infection Team, Public Health Scotland, Glasgow, UK

[4]School of Geosciences, The University of Edinburgh, Edinburgh, UK

[5]Department of Public Health, Environments and Society, London School of Hygiene & Tropical Medicine, London, UK

[6]Centre for Maternal and Child Health Research, City, University of London, London, UK

[7]Faculty of Built Environment, Tampere University, Tampere, Finland

[8]Centre for Inflammation Research, University of Edinburgh, Edinburgh, UK

[2,9]Centre for Clinical Brain Sciences, University of Edinburgh, Edinburgh, UK

**Joint last authors

†Corresponding author. Address for correspondence: [1]Population, Policy and Practice Research and Teaching Department, University College London Institute of Child Health, 30 Guilford Street, London WC1N 1EH, UK. p.hardelid@ucl.ac.uk

Confidential: For Review Only

Abstract

Background
There have been no population-based studies of SARS-CoV-2 testing, PCR-confirmed infections and COVID-19-related hospital admissions across the full paediatric age range. We examine the epidemiology of SARS-CoV-2 in children and young people (CYP) aged <23 years.

Methods
We used a birth cohort of all children born in Scotland since 1997, constructed via linkage between vital statistics, hospital records and SARS-CoV-2 surveillance data. We calculated risks of tests and PCR-confirmed infections per 1000 CYP-years between August and December 2020, and COVID-19-related hospital admissions per 100,000 CYP-years between February and December 2020. We used Poisson and Cox proportional hazards regression models to determine risk factors.

Results
Among the 1226855 CYP in the cohort, there were 378,402 tests, 19,005 PCR confirmed infections and 346 admissions, corresponding to rates of 770.8/1000 (95% confidence interval 768.4-773.3), 179.4/1000 (176.9-182.0) and 29.4/100,000 (26.3-32.8) CYP-years respectively. Infants had the highest COVID-19-related admission rates. Chronic conditions, particularly multiple types of conditions, was strongly associated with COVID-19-related admissions across all ages. Overall, 49% of admitted CYP had at least one chronic condition recorded.

Conclusions
Infants, and CYP with chronic conditions are at highest risk of admission with COVID-19. Half of admitted CYP had chronic conditions. Studies examining COVID vaccine effectiveness among children with chronic conditions, and whether maternal vaccine during pregnancy prevents COVID-19 admissions in infants are urgently needed.

Confidential: For Review Only

What is already known on this topic

Children are less likely to suffer severe symptoms of SARS-CoV-2 infection than adults. There are few population-based studies of the epidemiology of SARS-CoV-2 in children not admitted to hospital.

What this study adds

Using a national birth cohort from Scotland during 2020, we found that children and young people with chronic conditions were more likely to be tested, but secondary school aged children with chronic conditions were less likely to have a confirmed infection. Infants and children/young people with chronic conditions were at highest risk of admission.

How this study might affect research, practice or policy

Studies examining COVID vaccine effectiveness among children with chronic conditions, and whether maternal vaccine during pregnancy prevents COVID-19 admissions in infants are urgently needed.

Confidential: For Review Only

Background

Children are much less likely to experience hospital admission and mortality related to severe acute respiratory syndrome coronavirus-2 (SARS-CoV-2) infection than adults.[1] In Europe in 2020, 1.7% of COVID-19-related hospital admissions were in children <19 years of age.[2] Over the course of the pandemic our understanding of how SARS-CoV-2 infection affects children has also improved. Children who experience more severe symptoms of SARS-CoV-2 may present with acute infection symptoms such as fever or cough[3-5] Other children may develop an acute inflammatory syndrome, Paediatric Inflammatory Syndrome Temporally associated with SARS-CoV-2 (PIMS-TS; also referred to as Multisystem Inflammatory Syndrome related to COVID; MIS-C), several weeks after initial infection.[6-8] Children aged <2 years old appear to be over-represented among children admitted to hospital with acute symptoms, whereas children aged 10 years or older account for the largest proportion of admitted PIMS-TS cases.[4 9]

Among children admitted to hospital with SARS-CoV-2 or PIMS-TS, those with specific chronic respiratory, neurological, gastrointestinal or cardiovascular conditions, and particularly children with multiple comorbidities, were at increased risk of Paediatric Intensive Care Unit (PICU) admission or death. Infants and teenagers appeared to have higher odds of these severe outcomes compared to children aged 1-4 years old.[10 11] A lower reported risk of severe disease and, until 2021, relatively lower rates of infection in children, have supported a narrative that the benefits and risks (primarily of myocarditis following second dose mRNA vaccines in young men[12 13]) of vaccinations in children are finely balanced.

Most studies of paediatric SARS-CoV-2 infection have been case series of infected or hospitalised children, making calculations of population-based risks of confirmed infections and associated admissions among different groups of children, including children with chronic conditions, impossible. Our aim was to provide population-based estimates of risk of SARS-CoV-2 testing, polymerase chain reaction (PCR)-confirmed infections and COVID-19 related admissions in children and young people (CYP) based on age, presence of chronic conditions, and socioeconomic status that could support vaccination and other policy recommendations across the whole paediatric population.

Methods

Data sources

We used a national birth cohort of all CYP born in Scotland from 1997 onwards, developed from administrative health datasets linked to public health surveillance data on SARS-CoV-2

Confidential: For Review Only

test results, originally constructed for the PICNIC study.[14] Birth registrations comprised the cohort spine, and CYP are linked over time and between databases using the Community Health Index number, a unique personal identifier recorded at all interactions with the Scottish National Health Service (NHS). Table 1 summarises the databases and variables used in this study.

Confidential: For Review Only

Table 1. Datasets and variables from the national Scottish birth cohort used in the study

Dataset	Dataset details	Variables used
National Records for Scotland (NRS) birth registrations	Vital registration data on all children born in Scotland and their parents, collected via registry offices	Week and year of birth; Baby Sex; Socio-economic position (parents' occupation at birth).
Scottish Morbidity Record (SMR)-01	Contains data on post-neonatal admissions and day cases to all NHS hospitals in Scotland	Admission and discharges dates; Primary and secondary diagnoses during admission; Type of hospital admission; Admission and discharge data from Intensive Care Unit (ICU).
SMR-02 (maternity records)	Contains data on all maternity admissions (including deliveries) in Scotland	Estimated gestational age; birth weight; number of older siblings (parity).
COVID-19 Tests	Contains data on all PCR- and antigen tests for SARS-Cov-2 with results and dates	Date of testing; Type of test; Result.
National Records for Scotland (NRS) death registrations	Vital registration data on children who died in Scotland	Date of death; Cause of death.
Scottish Birth Records (SBR)	Contains data on all children born in NHS hospitals, with data on neonatal admissions in and after April 2003	Diagnoses recorded at or shortly after birth ; Primary and secondary diagnoses at birth admission.
CHI register	Contains data on migration in/out Scotland	Migration outside Scotland.
Child Health Surveillance Programme-School	Contains data school health visits	Height and weight at age 5

Confidential: For Review Only

Study population and follow-up

We included CYP born in Scotland from 1st April 1997 to 31st December 2020. Children born at less than 24 weeks' gestation or with a birthweight <500 grammes were excluded,[15] as were CYP whose mothers were not resident in Scotland at the time of delivery, and CYP who migrated out of Scotland before 1st February 2020. For analyses of SARS-CoV-2 tests and positive test results (from now on referred to as PCR-confirmed infections), CYP were followed from birth or 1st August 2020 (whichever occurred last), until death, migration from Scotland, their 23rd birthday or 31st December 2020, whichever occurred first. 1st August 2020 was chosen as the follow-up start date for analyses of tests and PCR-confirmed infections since this is when testing for SARS-CoV-2 became commonly available in the community (rather than solely in hospitals) for children of all ages.[16] For calculation and analyses of rates of COVID-19-related admissions, we used 1st February 2020 as the follow-up start date. This allowed us to include all COVID-19-related hospital admissions since the start of the pandemic.

Outcomes

Our primary outcomes were rates of SARS-CoV-2 PCR tests (positive or negative); PCR-confirmed SARS-CoV-2 infections; COVID19-related hospital admissions. Our secondary outcomes were PIMS-TS admissions and COVID19-related intensive care unit (ICU) stays. Supplementary Text 1 details how each of these outcomes were derived.

Risk factors

We examined four risk key factors for testing, confirmed infections and hospital admission outcomes: age group, sex, family socio-economic position and history of chronic conditions. Age as of 1st February 2020 was grouped into: <1 year (this also includes children born during 2020), 1-4 years, 5-11 years, 12-17 years and 18-22 years. We chose these age groups to reflect likely mixing patterns based on age (i.e. prior to formal childcare, nursery/preschool, primary school, secondary school, and higher/further education or work). Family socio-economic position was defined using parents' (father's, or mother's if the birth was not jointly registered) occupation recorded on birth registration, coded using the UK National Statistics Socio-economic Classification (NS-SEC).[17] We collapsed the NS-SEC classes into: high (managerial and professional occupations), middle (intermediate occupations) and low (routine and manual occupations) socio-economic position. We identified history of chronic conditions by examining International Classification of Disease version 10 (ICD-10) diagnostic codes recorded in the Scottish Morbidity Record (SMR-01)

https://mc.manuscriptcentral.com/bmjpo

between 1 Jan 2015 and 31 January 2020. using an existing code list.[18] For children aged less than five years at the start February 2020 or born during 2020, we used all available SMR-01 data and any diagnoses recorded on Scottish Birth Records (SBR). Chronic conditions were classified into 8 types: developmental/mental health, blood/cancer, chronic infections, respiratory, metabolic/gastrointestinal/endocrine/genitourinary, musculoskeletal/skin neurological/sensory, and cardiac conditions. These were further grouped into none, one type of condition, and more than one type of chronic condition for analyses.

We further explored whether gestational age and the number of older siblings affected PCR confirmed infection and hospital admission risk in children aged <5 years, and Body Mass Index (BMI) in CYP aged 5-17. Gestational age was grouped as: preterm (<37 weeks) and term/late term (≥37 weeks). Number of older siblings (indicated by parity) was grouped as: no older siblings, one older sibling and two or more older siblings. BMI was derived from the Child Health Surveillance Programme -School dataset collected from children starting their first year at school (at age 5 years), and categorised[19] as underweight (<5th percentile), healthy weight (5th to <85th percentile) and overweight/obese (≥85th percentile).

Statistical analyses

We calculated rates of testing and PCR confirmed infections per 1,000 CYP years and hospital admission per 100,000 CYP-years with 95% confidence intervals stratified by each risk factors. We estimated the median length of stay with interquartile ranges (IQRs) for COVID-19 related hospital admissions. We calculated the proportion of children with a COVID-19 related who had a chronic condition recorded either at baseline, or during the COVID-19 admission.

We examined the association between risk factors and testing rates using Poisson regression models with robust standard errors to account for multiple tests per child. To examine the association between risk factors and PCR-confirmed SARS-CoV-2 infection, and COVID19-related admission risk we used Cox proportional hazards regression models. Where a child had multiple COVID-19-related admissions, only the first was included in the Cox proportional hazards models. For each primary outcome, we first fitted an overall model including all ages and age group, sex, socio-economic position and history of chronic conditions as risk factors. We tested for interaction with age group and each of the other main risk factors using the Wald test. Two-sided *p*-values <0.05 were considered statistically significant.

https://mc.manuscriptcentral.com/bmjpo

We then fitted models for each primary outcome stratified by age group if a statistically significant interaction with age was identified for any of the other variables or if we identified non-proportional hazards. In further analyses for ages <5 years old we included parity and gestational age as additional risk factors in the models; and for ages 5-17 years we included BMI category. We tested the proportional hazards assumption of the Cox model by inspecting plots of Schoenfeld residuals[20] and survival curves according to each main risk factor.

As there was only a small number of events for our secondary outcomes, we report the number of cases, median length of stay and age (with IQRs) only. All analyses were based on complete cases, as only a small number of CYP were missing values for any of the main variables. All statistical analyses were performed using Stata 16.0.

Sensitivity analyses

We examined the number of COVID-19 hospital admissions that were identified as occurring up to 14 days after a positive SARS-CoV-2 test. We repeated the analyses for hospital admission risk using a more specific definition of a COVID-19-related admission restricted to emergency admissions with an ICD-10 code indicating COVID-19 (U07.1 or U07.2)[21] as the primary diagnosis.

Patient and public involvement

The PICNIC study has been presented to a number of parent groups, including the Great Ormond Street Hospital Biomedical Research Centre Parents and Carers Advisory Group, and a coffee morning for parents at Shelter's Birmingham Office. This COVID epidemiology substudy has not been specifically reviewed by parents.

Results

This study included 1,226,855 CYP (Supplementary Figure 1). The median age in February 2020 was 11 years (IQR 5-17), and 8.0% of the cohort (97,884/1,226,855 CYP) had at least one chronic condition recorded in their hospital or birth record in the previous five years (Supplementary Table 1).

SARS-CoV-2 testing

Between 1st August (week 31) to 31st December 2020 (week 52) we identified 378,402 PCR tests linked to 256,741 CYP; hence 20.9% of CYP in the cohort had at least one test. Supplementary Figure 2 shows the weekly number of PCR tests by age group. The crude

Confidential: For Review Only

testing rate was 770.8 (95%CI 768.4-773.3) per 1,000 CYP-years. The majority of CYP had been tested only once (200,288; 78.0%); 40,188 (15.7%) had been tested twice and 16,265 (6.3%) more than twice. Further results regarding rates of testing by week and risk factor can be found in Supplementary Text 2 and Supplementary Tables 2, 3,4 and 5.

PCR-confirmed infections

Among the 378,402 PCR tests identified in the cohort, 20,003 (5.3%) were positive and 7,275 (1.9%) were void. Excluding multiple positive tests per CYP, this corresponds to 19,005 PCR confirmed index infections in 7.4% (19,005/ 256,741) of the CYP who were tested between 1st August 2020 and 31st December 2020.

The overall rate of PCR-confirmed infections was 179.4 (95% CI 176.9-182.0) per 1,000 CYP-years. Young adults (aged 18-22 years) had the highest rates of PCR-confirmed infections and those aged 1-4 years the lowest (Table 2). Infants had the highest PCR-confirmed infection rates among preschool children, otherwise infection rates were positively correlated with age. CYP with chronic conditions had a lower risk of PCR-confirmed infection, particularly among secondary school aged children (Supplementary Table 4 & Table 3). Age-group specific analyses showed that among preschool children, PCR-confirmed infection rates were higher among children from lower socio-economic backgrounds, whereas the opposite was observed among CYP aged 12 years and above (Table 3).

Confidential: For Review Only

Table 2. Rates of PCR confirmed infections (per 1,000 CYP-years) by age group, sex, socio-economic position, and history of chronic conditions

	Age<1 year			Age 1-4 years			Age 5-11 years			Age 12-17 years			Age 18-22 years		
	Events	Rate	95%CI	Events	Rate	95%CI	Events	Rate	95%CI	Events	Rate	95%CI	Events	Rate	95%CI
	223	80	70, 91	1136	62	58, 65	3039	96	93, 100	4929	193	188, 198	9687	350	344, 358
SEX															
Male	121	79	66, 94	595	60	55, 65	1545	91	87, 96	2313	179	172, 187	4487	365	355, 376
Female	102	81	67, 98	541	64	59, 69	1494	102	97, 108	2616	207	199, 215	5200	338	329, 348
SOCIO-ECONOMIC POSITION															
High	21	51	33, 78	141	51	43, 60	292	81	73, 91	503	175	161, 191	1181	481	455, 510
Middle	111	83	69, 100	571	63	58, 69	1446	102	97, 108	2231	202	194, 210	5510	353	344, 363
Low	91	88	71, 108	424	64	58, 70	1301	94	89, 100	2195	189	181, 197	2996	312	301, 324
CHRONIC CONDITIONS															
None	202	81	71, 94	1031	63	60, 67	2757	98	94, 102	4591	199	194, 205	8766	366	358, 373
One	10	44	24, 82	77	45	36, 57	225	87	76, 99	272	143	127, 161	735	267	249, 287
>One	11	130	72, 235	28	54	37, 78	57	74	57, 95	66	107	84, 136	186	202	175, 233

PCR, Polymerase Chain Reaction; CYP, Children and Young Person

Confidential: For Review Only

Table 3 Time to PCR confirmed infection: hazard ratios (HR) by age group mutually adjusted for sex, socio-economic position and history of chronic conditions

	Age <1 years		Age 1-4 years		Age 5-11 years		Age 12-17 years		Age 18-22 years	
Number of CYP in model	9661		49288		70245		76262		70212	
Number of PCR confirmed infections	276		1114		1286		2706		4338	
	AdjHR	95%CI	AdjHR	95%CI	AdjHR	95%CI	AdjHR	95%CI	AdjHR	95%CI
SEX										
Male	1	-	1	-	1	-	1	-	1	-
Female	1.15	0.93, 1.41	1.08	0.97, 1.20	1.10	1.02, 1.19	1.20	1.15, 1.27	0.96	0.92, 1.00
SOCIO-ECONOMIC POSITION										
High	1	-	1	-	1	-	1	-	1	-
Middle	1.44	1.02, 2.04	1.30	1.09, 1.55	1.26	1.10, 1.44	1.02	0.94, 1.11	0.75	0.70, 0.80
Low	1.46	1.02, 2.08	1.31	1.10, 1.58	1.17	1.02, 1.34	0.91	0.83, 0.98	0.66	0.62, 0.71
CHRONIC CONDITIONS										
None	1	-	1	-	1	-	1	-	1	-
One	0.62	0.39, 0.98	0.74	0.60, 0.91	0.87	0.75, 1.00	0.79	0.71, 0.88	0.76	0.70, 0.82
> One	1.23	0.72, 2.10	0.79	0.55, 1.12	0.76	0.58, 1.00	0.59	0.48, 0.73	0.58	0.50, 0.67

PCR, Polymerase Chain Reaction; Adj HR, Adjusted Hazard Ratio; CI, Confidence Intervals.

Confidential: For Review Only

Children aged <5 years with one older sibling had a reduced risk of a PCR confirmed infection compared to children with no older siblings (Supplementary Table 6 and 7). Further, in children aged 12-17 years, being overweight/obese increased the risk of a PCR-confirmed infection compared to being of normal BMI.

COVID19-related-hospital admissions

Between 1st February 2020 and 31 December 2020, there were 81,312 admissions in 55,940 CYP. 346 (0.6%) admissions in 318 CYP were identified as COVID-19-related. The median length of stay was 2 days (IQR 1-4 days). There were 110 admissions between February and July (31.8%) and 236 (68.2%) between August and December (Figure 1). 49.4% (*n*=157) of the 318 CYP admitted had at least one type of chronic condition recorded; and 23.3% (74 of 318; 46.5% of the 159 children with at least one chronic condition) had multiple types of chronic conditions recorded.

The overall COVID-19 related admission rate was 29.4/100,000 (95%CI 26.3-32.8) CYP years (Table 4). Infants had the highest COVID-19 related admission rate: 120.6/100,000 (95%CI 92.2-157.9). CYP with chronic conditions, and particularly children with more than one chronic condition type recorded, had the highest admission rates across all age groups.

Of the CYP with chronic conditions who had a COVID-19 related admission, neurological/sensory conditions were the common condition type recorded among children aged<12 years, whereas among those aged 12-22 years the most common conditions were developmental/mental health conditions.

Presence of one or more chronic condition significantly increased the risk of COVID-19-related admissions across all ages (Supplementary table 4). In age-stratified analyses, presence of a chronic condition remained the only statistically significant risk factor for COVID-19-related admission across all age groups (Table 5). We did not identify any statistically significant associations between prematurity, number of older siblings, or BMI category and COVID-19 related hospital admission risk (Supplementary Table 9), however the number of hospital admissions was low in this study.

https://mc.manuscriptcentral.com/bmjpo

Table 4. Rates of COVID-related admissions (per 100,000CYP-years) by age group, sex, socio-economic position, and history of chronic conditions

	Age<1 year			Age 1-4 years			Age 5-11 years			Age 12-17 years			Age 18-22 years		
	Events	Rate	95%CI	Events	Rate	95%CI	Events	Rate	95%CI	Even ts	Rate	95%CI	Events	Rate	95%CI
	53	121	92, 158	51	27	21, 36	55	16	12, 21	49	18	13, 23	110	49	41, 67
SEX															
Male	30	133	93, 190	22	23	15, 35	31	17	12, 25	26	18	12, 27	43	38	28, 55
Female	23	107	71, 162	29	32	22, 46	24	14	9, 21	23	17	11, 25	67	61	48, 91
SOCIO-ECONOMIC POSITION															
High	*	73	27, 194	*	18	7, 48	*	12	5, 29	*	9	3, 27	6	34	15, 75
Middle	*	118	80, 175	*	24	16, 36	*	15	10, 22	*	18	12, 28	55	43	33, 69
Low	*	139	93, 207	*	34	23, 50	*	18	12, 26	*	19	13, 29	49	62	47, 86
CHRONIC CONDITIONS															
None	*	105	78,142	*	20	15, 29	21	7	4,10	19	7	5,11	42	21	16, 28
One	*	277	115,666	*	38	16, 92	19	93	59, 146	17	113	70, 181	37	197	143, 272
>One	*	1032	387,2749	*	374	207, 675	15	326	196, 540	13	332	193, 573	31	547	385, 778

PCR, Polymerase Chain Reaction; CYP, Children and Young Person

*Redacted due to small numbers in some groups

Confidential: For Review Only

Table 5 Time-to-COVID related admissions: hazard ratios (HR) by age group mutually adjusted for sex, socio-economic position and history of chronic conditions

	Age <1 year		Age 1-4 years		Age 5-11 years		Age 12-17 years		Age 18-22 years	
Number of CYP in model	92,530		251,884		347,542		385,664		268,467	
N Admissions	53		51		55		49		110	
	AdjHR	95%CI	AdjHR	95%CI	AdjHR	95%CI	AdjHR	95%CI	AdjHR	95%CI
SEX										
Male	1	-	1	-	1	-	1	-	1	-
Female	0.82	0.51, 1.30	1.49	0.88, 2.51	1.10	0.62, 1.96	1.05	0.67, 1.67	1.47	0.99, 2.17
Socio-economic position										
High	1	-	1	-	1	-	1	-	1	-
Middle	1.70	0.66, 4.36	1.39	0.48, 4.03	0.91	0.34, 2.41	3.05	0.94, 9.92	1.03	0.44, 2.39
Low	2.07	0.81, 5.28	1.97	0.69, 5.61	0.92	0.35, 2.43	2.75	0.85, 8.93	1.27	0.54, 2.99
CHRONIC CONDITIONS										
None	1	-	1	-	1	-	1	-	1	-
One	3.14	1.50, 6.58	2.46	1.10, 5.53	13.73	6.99, 26.96	13.85	8.12, 23.61	8.86	5.62, 13.96
More than one	10.99	4.74, 25.48	19.71	10.45, 37.19	49.81	24.30, 102.12	40.96	22.89, 73.30	25.39	15.80, 40.82

Adj HR, Adjusted Hazard Ratio; CI, Confidence Intervals.

Confidential: For Review Only

ICU admissions and PIMS-TS cases

Thirteen (3.8%) of the 346 COVID-related admissions involved an ICU attendance, accounting for 1.2% of the 1,238 ICU admissions in CYP during the study period. The vast majority of these admissions were in CYP with a history of one or more types of chronic conditions. The median age of CYP admitted to ICU was 14 years (IQR 9-19 years) and the median length of stay at ICU was 6 days (IQR 2-7 days).

We identified less than five admissions with a diagnosis suggestive of PIMS-TS and temporally associated with a positive PCR test (<28 days prior admission by definition), all in males with an age spanning from 9 to 14 years. The median length of stay at admission was 10 days (IQR 6-14).

Sensitivity analyses

Of the 346 COVID-19-related admissions 203 (58.7%) had a specific COVID-19 ICD-10 code as the primary diagnosis and 258 (74.6%) were temporally associated with a SARS-CoV-2 positive test (Figure 2). Using the more specific definition of a COVID-19 related-admission the admission rates were 107.0 (95% CI 80.4-142.4), 13.4 (9.0-19.8), 8.0 (5.6 – 11.7), 9.3 (6.3-13.6) and 27.7 (21.6-35.5) per 100,000 CYP years in age groups <1, 1-4, 5-11, 12-17 and 18-22 years respectively (Supplementary Table 10 &11). This was between 12% (in infants) and 50% (in children aged 5-11 years) lower than the more inclusive definition used in the main analyses. Presence of one or more chronic conditions remained the only significant risk factor for hospital admission with the specific definition (Supplementary Tables 12 & 13) across the age groups. The median length of stay remained 2 days (IQR 1-5).

Discussion

Over one fifth of CYP in Scotland had at least one SARS-CoV-2 PCR test during 2020, and 1.5% had a PCR-confirmed infection. CYP with chronic conditions were more likely to be tested, but secondary school aged CYP with chronic conditions were less likely to have a PCR confirmed infection. Whilst COVID-19 related hospital admissions were uncommon (less than 3 per 10,000 CYP admitted in 2020), infants and CYP with chronic conditions recorded had the highest COVID-19 related-admission rates.

The Scottish data linkage infrastructure allowed us to include data for all CYP born in Scotland since 1997, thereby minimising selection bias and loss to follow-up. We relied on linkage between hospital admission and public health surveillance data to define COVID-19-

https://mc.manuscriptcentral.com/bmjpo

related admissions, allowing us to examine the robustness of our definitions in the linked data, rather than only relying on time difference between SARS-CoV-2 positive test and hospital admission alone. By using a national birth cohort for this study, we examined variations in population-based rates of SARS-CoV-2 testing, PCR confirmed infections and COVID-19-related hospital admissions across the full CYP age range, rather than only examining risk factors for ICU admission and death in hospitalised children.[22]

This study included data from the first year of the pandemic, when wildtype (until November 2020), followed by Alpha (dominant from December 2020) SARS-CoV-2 variants were circulating in Scotland. This study will need to be repeated to examine the impact of later circulating variants, including Delta and Omicron, and changing transmission dynamics as vaccination of adults appear to be concentrating virus circulation among younger age groups.[23] The UK roll out of COVID vaccination for children aged 12-15 years, started in July 2021,[24] is likely to change risks of hospital admission, particularly among children with chronic conditions. Further studies will need to examine whether the COVID-19 vaccination programme has amended the admission risks reported in this study. The results reported here provide baseline risks during the first pandemic year against which more recent data can be compared. Updates of our analyses are planned.

We based our classification of chronic conditions on coded information in SMR-01 and SBR records and may have missed some conditions that are primarily managed in primary or community care settings. Despite the use of a national birth cohort, the number of children admitted to hospital with SARS-CoV-2 during 2020 was small, therefore we were unable to estimate admission risks in groups of children with specific chronic conditions. Our classification of socio-economic position was based on parental occupation derived from birth certificates, which may not reflect current socio-economic circumstances (e.g.in older CYP).

Infants had the highest admission risk. A systematic review has indicated that infants are also at highest risk of requiring PICU admission once in hospital with COVID-19 disease.[25] However, admission rates in infancy related to SARS-CoV-2 (1/1000 child-years) during 2020 were lower than admission rates associated with confirmed influenza (2/1000 child-years)[26] or respiratory syncytial virus infections (22/1000-child years).[27] Future research should examine how COVID-19 vaccination programmes for pregnant women and older children, and removal of non-pharmaceutical interventions to control population mixing, affect infant SARS-CoV-2 admission rates.

Confidential: For Review Only

We demonstrated that a history of chronic conditions, and particularly living with multiple different types of chronic conditions, was the most prominent risk factor for COVID-19 related hospital admission rates among CYP. Further, CYP with chronic conditions were more likely to be tested than those without, but less likely to have a PCR confirmed infection. This may reflect lower threshold for testing among high-risk groups.

Pre- and primary school children from lower socio-economic backgrounds had higher risks of PCR-confirmed infection than children from higher socio-economic backgrounds. Younger children spend more time in the home with their parents, thus their risk of infection is therefore more strongly associated with their parents' occupation (and ability to work from home). In older CYP, we instead identified higher PCR-confirmed infection rates among children of higher socio-economic position, despite lower testing rates. This may be due to CYP from lower socio-economic position groups being less likely to attend post-16 education, including university. Large outbreaks occurred in universities in Scotland in the autumn of 2020, which led to a surge in case numbers in 18-22 year olds.[28] Linkage between SARS-CoV-2 test results, hospital admission and education data are required to confirm whether exposure in education settings can explain these differences in infection risk.

We did not find a statistically significant association between socio-economic position and risk of COVID-related admission. This is unlike some previous reports which have demonstrated higher all-age hospital admission rates in areas with higher area-level deprivation scores.[29] However, across all ages the vast majority of COVID-19 related admissions are in adults. As COVID-19 related admission rates in children are much lower than in adults, systematic differences in admission rates by socio-economic position among specific age groups of CYP are harder to detect, even when using national data. Further, as we used parental occupation to indicate socio-economic background, this may not reflect current socio-economic circumstances, as discussed above.

Our results showing that COVID-19 related admission rates in CYP peak in infancy indicates that further research and efforts to prevent COVID-19 admissions in children should include a focus on this age group. Pregnant women in Scotland are recommended to receive two doses of Pfizer/BioNTech COVID-19 vaccine. [30] As for pertussis[31 32] and influenza,[33 34] maternal vaccination during pregnancy could protect young babies from SARS-CoV-2 infection, however no studies to date have examined this. Further, given that CYP with chronic conditions are more likely to be admitted to hospital admission with COVID-19 disease than other CYP, studies monitoring the effectiveness of COVID-19 vaccines against

Confidential: For Review Only

severe outcomes in these high-risk groups are required to determine whether vaccination reduces the risk of admission.

We identified a peak in COVID-19-related hospital admissions in infants, and presence of chronic conditions as the strongest risk factor for hospital admissions in CYP, yet half of CYP admitted did not have any chronic conditions recorded. Further studies are urgently needed to examine whether maternal vaccine during pregnancy prevents COVID-19 admissions in infants. These data also provide baseline risks of infection and hospital admission for risk-benefit assessments of childhood vaccination, particularly for preschool children.

Confidential: For Review Only

Ethics statement

This study was approved by the University of Edinburgh School of Geosciences Ethics Committee (reference number 2020-401) and the Public Benefit and Privacy Panel for Health and Social Care (reference 1819-0049).

Funding:

This work was supported by UKRI-Medical Research Council, grant number MR/T016558/1

Acknowledgements

We are grateful to Professor Bianca De Stavola (UCL) for her advice on statistical modelling. The authors would like to acknowledge the support of the eDRIS Team (Public Health Scotland), and particularly Diane Rennie, for their involvement in obtaining approvals, provisioning and linking data and the use of the secure analytical platform within the National Safe Haven. This work uses data provided by patients and collected by the NHS as part of their care and support

Competing interests

None

Author contributions

PH conceived the study together with RW and SC. PH acquired the data supported by RW. GF and LW analysed the data. PH, GF and LW drafted the paper. All other authors read and commented on the paper. All authors have read and approved the final version.

Confidential: For Review Only

Figure legends

Figure 1 Monthly number of COVID-related hospital admissions (week 5-52, year 2020; 1st February 2020 to 31st December 2020)

Figure 2 Number of COVID-related admissions temporally associated with PCR positive test up to 14 days before admission and by primary and secondary COVID19 diagnosis (U07.1/U07.2) Total admissions (*n*)= 346

Confidential: For Review Only

References

1. Davies NG, Klepac P, Liu Y, et al. Age-dependent effects in the transmission and control of COVID-19 epidemics. *Nature Medicine* 2020;26(8):1205-11. doi: 10.1038/s41591-020-0962-9
2. European Centre for Disease Prevention and Control. COVID-19 in children and the role of school settings in transmission - first update. 2020 https://www.ecdc.europa.eu/sites/default/files/documents/COVID-19-in-children-and-the-role-of-school-settings-in-transmission-first-update_1.pdf Accessed: 02/03/2021
3. Swann OV, Holden KA, Turtle L, et al. Clinical characteristics of children and young people admitted to hospital with covid-19 in United Kingdom: prospective multicentre observational cohort study. *BMJ* 2020;370:m3249. doi: 10.1136/bmj.m3249
4. Götzinger F, Santiago-García B, Noguera-Julián A, et al. COVID-19 in children and adolescents in Europe: a multinational, multicentre cohort study. *The Lancet Child & Adolescent Health* 2020;4(9):653-61. doi: 10.1016/S2352-4642(20)30177-2
5. Irfan O, Muttalib F, Tang K, et al. Clinical characteristics, treatment and outcomes of paediatric COVID-19: a systematic review and meta-analysis. *Archives of Disease in Childhood* 2021;106(5):440-48. doi: 10.1136/archdischild-2020-321385
6. Toubiana J, Levy C, Allali S, et al. Association between SARS-CoV-2 infection and Kawasaki-like multisystem inflammatory syndrome: a retrospective matched case–control study, Paris, France, April to May 2020. *Eurosurveillance* 2020;25(48):2001813. doi: doi:https://doi.org/10.2807/1560-7917.ES.2020.25.48.2001813
7. Belot A, Antona D, Renolleau S, et al. SARS-CoV-2-related paediatric inflammatory multisystem syndrome, an epidemiological study, France, 1 March to 17 May 2020. *Eurosurveillance* 2020;25(22):2001010. doi: doi:https://doi.org/10.2807/1560-7917.ES.2020.25.22.2001010
8. Whittaker E, Bamford A, Kenny J, et al. Clinical Characteristics of 58 Children With a Pediatric Inflammatory Multisystem Syndrome Temporally Associated With SARS-CoV-2. *JAMA* 2020;324(3):259-69. doi: 10.1001/jama.2020.10369
9. Flood J, Shingleton J, Bennett E, et al. Paediatric multisystem inflammatory syndrome temporally associated with SARS-CoV-2 (PIMS-TS): Prospective, national surveillance, United Kingdom and Ireland, 2020. *The Lancet Regional Health – Europe* 2021;3 doi: 10.1016/j.lanepe.2021.100075
10. Harwood R, Yan H, Da Camara NT, et al. Which children and young people are at higher risk of severe disease and death after SARS-CoV-2 infection: a systematic review and individual patient meta-analysis. *medRxiv* 2021:2021.06.30.21259763. doi: 10.1101/2021.06.30.21259763
11. Ward JL, Harwood R, Smith C, et al. Risk factors for intensive care admission and death amongst children and young people admitted to hospital with COVID-19 and PIMS-TS in England during the first pandemic year. *medRxiv* 2021:2021.07.01.21259785. doi: 10.1101/2021.07.01.21259785
12. Witberg G, Barda N, Hoss S, et al. Myocarditis after Covid-19 Vaccination in a Large Health Care Organization. *New England Journal of Medicine* 2021;385(23):2132-39. doi: 10.1056/NEJMoa2110737
13. Simone A, Herald J, Chen A, et al. Acute Myocarditis Following COVID-19 mRNA Vaccination in Adults Aged 18 Years or Older. *JAMA Internal Medicine* 2021;181(12):1668-70. doi: 10.1001/jamainternmed.2021.5511
14. Favarato G, Clemens T, Cunningham S, et al. Air Pollution, housing and respiratory tract Infections in Children: NatIonal birth Cohort study (PICNIC): study protocol. *BMJ Open* 2021;11(5):e048038. doi: 10.1136/bmjopen-2020-048038
15. Hardelid P, Verfuerden M, McMenamin J, et al. The contribution of child, family and health service factors to respiratory syncytial virus (RSV) hospital admissions in the first 3 years of life: birth cohort study in Scotland, 2009 to 2015. *Eurosurveillance*

Confidential: For Review Only

2019;24(1):1800046. doi: doi:https://doi.org/10.2807/1560-7917.ES.2019.24.1.1800046
16. Fenton L, Gribben C, Caldwell D, et al. Risk of hospital admission with covid-19 among teachers compared with healthcare workers and other adults of working age in Scotland, March 2020 to July 2021: population based case-control study. *BMJ* 2021;374:n2060. doi: 10.1136/bmj.n2060
17. Office for National Statistics. The National Statistics Socio-economic classification (NS-SEC); 2010 https://www.ons.gov.uk/methodology/classificationsandstandards/otherclassifications/thenationalstatisticssocioeconomicclassificationnssecrebasedonsoc2010
18. Hardelid P, Davey J, Dattani N, et al. Child deaths due to injury in the four UK countries: a time trends study from 1980 to 2010. *PLoS One* 2013;8(7):e68323. doi: 10.1371/journal.pone.0068323
19. Cole TJ, Freeman JV, Preece MA. British 1990 growth reference centiles for weight, height, body mass index and head circumference fitted by maximum penalized likelihood. *Stat Med* 1998;17(4):407-29.
20. SCHOENFELD D. Partial residuals for the proportional hazards regression model. *Biometrika* 1982;69(1):239-41. doi: 10.1093/biomet/69.1.239
21. Public Health Scotland. Scottish Clinical Coding Standards – ICD-10. 2020 https://www.isdscotland.org/Products-and-Services/Terminology-Services/Clinical-Coding-Guidelines/Docs/SCCS-24-April-2020.pdf Accessed:
22. Griffith GJ, Morris TT, Tudball MJ, et al. Collider bias undermines our understanding of COVID-19 disease risk and severity. *Nature Communications* 2020;11(1):5749. doi: 10.1038/s41467-020-19478-2
23. Public Health Scotland. COVID-19 Daily Dashboard; 2021 https://public.tableau.com/app/profile/phs.covid.19/viz/COVID-19DailyDashboard_15960160643010/Overview
24. Joint Committee of Vaccination and Immunisation. JCVI statement on COVID-19 vaccination of children and young people aged 12 to 17 years: 15 July 2021; 2021 https://www.gov.uk/government/publications/covid-19-vaccination-of-children-and-young-people-aged-12-to-17-years-jcvi-statement/jvci-statement-on-covid-19-vaccination-of-children-and-young-people-aged-12-to-17-years-15-july-2021
25. Harwood R, Yan H, Talawila Da Camara N, et al. Which children and young people are at higher risk of severe disease and death after hospitalisation with SARS-CoV-2 infection in children and young people: A systematic review and individual patient meta-analysis. *eClinicalMedicine* 2022;44 doi: 10.1016/j.eclinm.2022.101287
26. Hardelid P, Verfuerden M, McMenamin J, et al. Risk factors for admission to hospital with laboratory-confirmed influenza in young children: birth cohort study. *Eur Resp J* 2017;50(3) doi: 10.1183/13993003.00489-2017
27. Hardelid P, Verfuerden M, McMenamin J, et al. The contribution of child, family and health service factors to respiratory syncytial virus (RSV) hospital admissions in the first 3 years of life: birth cohort study in Scotland, 2009 to 2015. *Euro Surveill* 2019;24(1) doi: 10.2807/1560-7917.ES.2019.24.1.1800046
28. Public Health Scotland. COVID-19 in Scotland Daily Dashboard: Trends and demographics; 2021 https://public.tableau.com/app/profile/phs.covid.19/viz/COVID-19DailyDashboard_15960160643010/Overview
29. The Scottish Parliament. Health Inequality and COVID-19 in Scotland. 2021 https://digitalpublications.parliament.scot/ResearchBriefings/Report/2021/3/23/ee202c60-93ad-4a27-a6e7-67613856ba24 Accessed: 27/10/2021
30. UK Health Security Agency. Green Book Chapter 14a: COVID-192021.
31. Dabrera G, Amirthalingam G, Andrews N, et al. A Case-Control Study to Estimate the Effectiveness of Maternal Pertussis Vaccination in Protecting Newborn Infants in England and Wales, 2012–2013. *Clinical Infectious Diseases* 2014;60(3):333-37. doi: 10.1093/cid/ciu821

For peer review only

32. Sandmann F, Jit M, Andrews N, et al. Infant Hospitalizations and Fatalities Averted by the Maternal Pertussis Vaccination Program in England, 2012–2017: Post-implementation Economic Evaluation. *Clinical Infectious Diseases* 2020;71(8):1984-87. doi: 10.1093/cid/ciaa165
33. Benowitz I, Esposito DB, Gracey KD, et al. Influenza Vaccine Given to Pregnant Women Reduces Hospitalization Due to Influenza in Their Infants. *Clinical Infectious Diseases* 2010;51(12):1355-61. doi: 10.1086/657309
34. Mølgaard-Nielsen D, Fischer TK, Krause TG, et al. Effectiveness of maternal immunization with trivalent inactivated influenza vaccine in pregnant women and their infants. *Journal of Internal Medicine* 2019;286(4):469-80. doi: https://doi.org/10.1111/joim.12947

Confidential: For Review Only

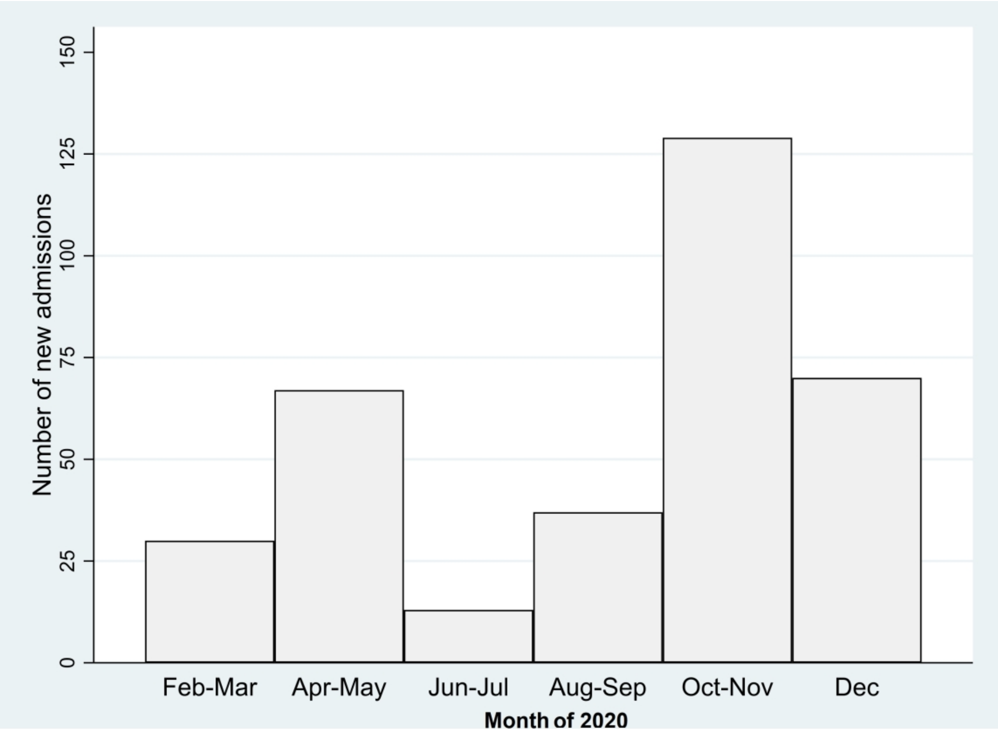

387x282mm (216 x 216 DPI)

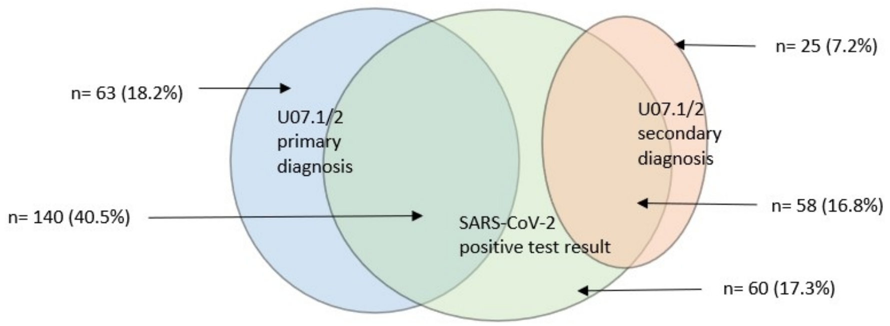

153x59mm (144 x 144 DPI)

SARS-CoV-2 tests, confirmed infections and hospital admissions in children and young people: birth cohort study

Supplementary material

Supplementary text 1

Outcome definitions

We included all SARS-CoV-2 PCR tests recorded between1st August 2020 and 31st December 2020 in the COVID19 Tests Dataset. The samples were collected in hospitals, primary care, via national testing centres, or self-collection via home test kits. We did not include antigen (lateral flow device) test results, as only 5% of test results in the cohort during the study period were from antigen tests. We defined as duplicate tests multiple tests taken on the same day, in the same CYP, with the same result, irrespective of whether they were taken at different locations. All duplicate tests, whether positive or negative, were excluded when calculating testing rates. A PCR confirmed infection was defined as the first record of a positive SARS-CoV-2 PCR test result (the index positive test) recorded in the COVID19 Tests dataset between1st August 2020 and 31st December 2020. Public Health Scotland recommends excluding all repeat positive tests within 90 days of the index positive sample date, and less than 5 CYP had multiple positive results beyond this time period. Therefore, only the first positive SARS-CoV-2 PCR test result for each child was included when calculating rates of PCR-confirmed SARS-CoV-2 infections.

We included all COVID-19-related hospital admissions between 1st February and 31st December 2020. To define COVID-19 related hospital admissions, we first linked episodes in the hospital admission dataset (Scottish Morbidity Record-01; Table 1) into admissions by assuming that episodes where the difference between the admission date and previous discharge date was ≤1 day[22] indicated the same admission. Second, we identified COVID-19 related admissions where: (i) an individual had tested positive for SARS-CoV-2 up to 14 days prior to hospital admission, on the day of admission, or in between the hospital admission and discharge date, and/or (ii) an International Classification of Diseases-version 10 (ICD-10) diagnostic code for COVID-19 (U07.1 – U07.2) had been recorded during an admission as a primary or secondary diagnosis.

https://mc.manuscriptcentral.com/bmjpo

Confidential: For Review Only

Since the ICD-10 code for PIMS-TS (U07.5) was introduced at the end of the follow-up period, we used other ICD-10 codes indicating systemic inflammatory response syndrome of infectious origin without organ failure (R65X), cardiogenic shock (R57X) or other specified systemic involvement of connective tissue (M35.8), suggestive of PIMS-TS recorded during an admission which had a positive SARS-CoV-2 PCR test within 28 day prior to the admission date.

A COVID-19-related intensive care unit (ICU) stay was defined where a child had an SMR-01 episode with 'significant facility' recorded with a positive SARS-CoV-2 PCR test to 21 days prior to the start of, or during, the ICU stay. ICU episodes where the difference between the ICU admission date and previous ICU discharge date was ≤1 day were assumed to indicate the same ICU stay.

Confidential: For Review Only

Supplementary text 2

Risk factors for testing by age

Testing rates varied by age group and chronic conditions; it was higher in children aged 1-4 years, young adults (age 18-22 years), and those with more than one chronic condition (Supplementary Table 2 & 3).

The all-age model suggested increasing age and chronic conditions were strongly associated with being tested (Supplementary Table 4). In age-group stratified analyses, a history of chronic conditions was strongly associated with higher testing rates (Supplementary Table 5), particularly among infants.

Confidential: For Review Only

Supplementary Tables

Supplementary TABLE 1: Cohort baseline characteristics (*n*=1,226,855)

	Number	%
Sex		
Male	628,410	51.2
Female	598,445	48.8
missing	*0*	*0*
Age (years)*		
Median 10.8 years (IQR 5-17) y		
<1 year	92,539	7.5
1-4 years	206,677	16.9
5-11 years	326,455	26.6
12-17 years	358,195	29.2
18-22 years	242,989	19.8
Missing	*0*	*0*
Socio economic position**		
High	136,938	11.2
Middle	582,342	47.5
Low	507,563	41.4
Missing	*12*	*0*
Chronic conditions***		
None	1,128,971	92.0
One	78,016	6.4
More than one type	19,868	1.6
Gestational age (weeks) (aged* <5yr, *n*=292,289)		
Pre-term (<37 weeks)	23,825	8.0
Normal/Post-term (≥37 weeks)	268,464	89.7
Missing	*6,927*	*2.3*
Number of older siblings (aged*<5yr, *n*=289,800)		
None	124,289	41.5
One	102,944	34.4
Two or more	62,567	20.9
Missing	*9,416*	3.2
BMI *** (aged* 5-17, *n*=550,874)		
Underweight	8,930	1.30
Normal	421,182	60.2
Overweight/Obese	120,762	17.6
missing	*142,776*	*20.9*

* As on 1st February 2020; aged<1yr includes those born between 1 Feburary2020 and 31 December 2020. ** From UK National Statistics Socio-economic Classification (NS-SEC): SEP (managerial and professional occupations), middle SEP (intermediate occupations), low SEP (routine and manual occupations). ***Includes any chronic conditions recorded in the hospital records in the previous five years. ***As recorded in the Child Health Surveillance Programme-School at aged 5 and standardised according to the British 1990 growth reference standards (Cole 1998): underweight (<5th percentile), normal weight (5th to <85th percentile), overweight/obese (≥85th percentile).

Confidential: For Review Only

Supplementary Table 2 Rate testing by age group (age 0-4 years) per 1,000 CYP-years

	**Age <1 year**				**Age 1-4 years**			
	Events	Rate	95%LCI	95%UCI	Events	Rate	95%LCI	95%UCI
Overall	9509	482	473	492	59176	702	696	708
SEX								
Male	5256	519	506	534	32284	743	735	752
Female	4253	443	430	457	26892	658	650	666
Socio-economic position								
High	1368	551	522	581	9417	937	918	956
Middle	4417	466	452	480	29119	729	720	737
Low	3724	481	466	497	20637	602	594	610
CHRONIC CONDITIONS								
None	7678	408	399	417	50790	657	651	663
One	980	1323	1242	1408	5712	997	971	1023
More than one	851	5481	5124	5861	2674	2082	2005	2163
GESTATIONAL AGE								
pre-term	8099	457	448	468	52447	693	687	699
Term/post-term	1207	797	753	843	5566	818	796	839
NUMBER OLDER SIBLINGS								
None	4030	479	464	494	26920	770	761	779
One	3182	489	472	506	20177	692	682	701
More than one	1996	487	466	509	10371	589	577	600
BMI								
underweight	-	-	-	-	-	-	-	-
normal	-	-	-	-	-	-	-	-
overweight/obese	-	-	-	-	-	-	-	-

Confidential: For Review Only

Supplementary Table 3 Rate of testing by age group (age 5-22 years) per 1,000 CYP-years

	**Age 5-11 years**				**Age 12-17 years**				**Age 18-22 years**			
	Events	Rate	95%LCI	95%UCI	Events	Rate	95%LCI	95%UCI	Events	Rate	95%LCI	95%UCI
Overall	92007	583	580	587	79771	623	619	627	137939	1364	1357	1371
SEX												
Male	49757	622	616	628	45322	586	581	592	46290	895	887	903
Female	42250	558	553	564	46831	635	630	641	91649	1854	1842	1866
Socio-economic position												
High	10528	591	579	603	10059	531	521	541	9961	1198	1175	1222
Middle	41274	593	587	600	39678	612	606	618	78068	1390	1380	1399
Low	40203	588	582	595	42416	631	625	637	49909	1362	1350	1374
CHRONIC CONDITIONS												
None	80747	552	549	556	70707	592	587	596	117695	1303	1296	1311
One	8000	850	832	869	6395	944	921	967	14918	1789	1761	1818
More than one	3260	1538	1486	1592	2669	1530	1473	1590	5326	2137	2080	2195
GESTATIONAL AGE												
pre-term	-	-	-	-	-	-	-	-	-	-	-	-
term	-	-	-	-	-	-	-	-	-	-	-	-
post-term	-	-	-	-	-	-	-	-	-	-	-	-
NUMBER OLDER SIBLINGS												
None	-	-	-	-	-	-	-	-	-	-	-	-
One	-	-	-	-	-	-	-	-	-	-	-	-
More than one	-	-	-	-	-	-	-	-	-	-	-	-
BMI												
underweight	1009	626	589	666	1250	644	610	681	-	-	-	-
normal	49281	567	562	572	46792	594	589	600	-	-	-	-
overweight/obese	15287	588	579	598	14415	639	628	649	-	-	-	-

Confidential: For Review Only

Supplementary Table 4 Results of models adjusted for age group, sex, socio-economic position, and history of chronic conditions

	Testing*		PCR confirmed infection**		Admission***	
	Adj IRR	95%CI	Adj HR	95%CI	Adj HR	95%CI
AGE GROUP						
<1year	0.94	0.92, 0.95	0.76	0.70, 0.84	10.11	7.14, 14.32
1-4 years	1.14	1.12, 1.15	0.58	0.54, 0.62	1.11	0.74, 1.68
5-11 years	1.00	-	1	-	1	-
12-17 years	1.15	1.13, 1.16	2.38	2.28, 2.48	1.25	0.87, 1.80
18-22 years	1.89	1.87, 1.91	3.13	3.00, 3.26	2.32	1.66, 3.23
SEX						
Male	1	-	1	-	1	-
Female	1.16	1.15, 1.17	1.05	1.02, 1.08	1.11	0.89, 1.39
Socio-economic position						
High	1	-	1	-	1	-
Middle	1.00	0.98, 1.01	0.95	0.91, 1.00	1.34	0.86, 2.11
Low	0.96	0.95, 0.98	0.85	0.81 0.89	1.53	0.98. 2.40
CHRONIC CONDITIONS						
None	1	-	1	-	1	-
One	1.38	1.36, 1.40	0.75	0.71, 0.79	7.55	5.79, 9.86
More than one	1.85	1.81, 1.88	0.61	0.55, 0.68	26.17	19.79, 34.59

PCR, Polymerase Chain Reaction; Adj IRR, Adjusted Incidence Risk Ratio; Adj HR, Adjusted Hazard Ratio; CI, Confidence Intervals.

Footnotes:

* Wald test for interaction: age and sex p<0.0001; age and socio-economic position p<0.0001; age and chronic conditions p<0.0001;

**interaction: age and sex p<0.0001 , age and socio-economic position p<0.0001 , age and chronic conditions p=0.0004; global test to check proportionality assumption p<0.0001 (for age, socio-economic position, chronic conditions).

*** interaction: age and sex p= 0.045 , age and NS-SEC p= 0.94 , age and chronic conditions p =0.26; global test to check proportionality assumption p 0.0009 (for age, chronic condition

Confidential: For Review Only

Supplementary Table 5 Incidence Risk Ratio of being tested by age group mutually adjusted for sex, socio-economic status, history of chronic conditions and parity and pre-term (age<5 years) and BMI (aged 5-17 years)

	**Age <1 year**			**Age 1-4 years**			**Age 5-11 years**			**Age 12-17 years**		
N CYP in model	89202			200590			310670			231202		
N tests in model	94799			213258			323020			247143		
	IRR	95%LCI	95%UCI	IRR	95%LCI	95%UCI	IRR	95%LCI	95%UCI	IRR	95%LCI	95%UCI
SEX												
Male	1.00	-	-	1.00	-	-	1.00	-	-	1.00	-	-
Female	0.89	0.86	0.92	0.91	0.90	0.93	0.91	0.89	0.92	1.15	1.13	1.17
SOCIO-ECONOMIC POSITION												
High	1.00	-	-	1.00	-	-	1.00	-	-	1.00	-	-
Middle	0.76	0.73	0.80	0.84	0.82	0.86	1.02	1.00	1.05	1.09	1.06	1.13
Low	0.62	0.60	0.65	0.76	0.74	0.78	1.01	0.98	1.03	1.10	1.06	1.13
CHRONIC CONDITIONS												
None	1.00	-	-	1.00	-	-	1.00	-	-	1.00	-	-
One	2.13	2.02	2.25	1.44	1.40	1.48	1.46	1.42	1.49	1.48	1.43	1.53
More than one	3.89	3.66	4.14	2.22	2.11	2.33	2.14	2.03	2.25	1.91	1.80	2.02
GESTATIONAL AGE												
Pre-term	1.10	1.04	1.15	1.07	1.04	1.10	-	-	-	-	-	-
Term/post-term	1.00	-	-	1.00	-	-	-	-	-	-	-	-
NUMBER OLDER SIBLINGS												
None	1.00	-	-	1.00	-	-	-	-	-	-	-	-
One	0.99	0.95	1.02	0.91	0.89	0.93	-	-	-	-	-	-
More than one	0.86	0.82	0.90	0.82	0.80	0.84	-	-	-	-	-	-
BMI												
underweight	-	-	-	-	-	-	1.04	0.98	1.11	1.10	1.02	1.19
normal	-	-	-	-	-	-	1.00	-	-	1.00	-	-
overweight/obese	-	-	-	-	-	-	1.03	1.01	1.04	1.05	1.03	1.08

Confidential: For Review Only

Supplementary Table 6 Rate of PCR confirmed infections by age group per 1,000 CYP-years – extra variables

	**Age<1 year**				**Age 1-4 years**			
	Events	Rate	95%LCI	95%UCI	Events	Rate	95%LCI	95%UCI
	223	80	70	91	1136	62	58	65
GESTATIONAL AGE								
pre-term	192	78	68	90	1022	62	59	66
term/post-term	24	85	57	126	89	53	43	66
NUMBER OLDER SIBLINGS								
None	109	92	76	110	541	65	60	71
One	58	60	46	77	358	57	51	63
More than one	48	86	65	114	201	61	53	70
	**Age 5-11 years**				**Age 12-17 years**			
	Events	Rate	95%LCI	95%UCI	Events	Rate	95%LCI	95%UCI
	3039	96	93	100	4929	193	188	198
BMI								
underweight	38	112	82	154	72	179	142	225
normal	1723	101	96	106	2791	182	175	189
overweight/obese	545	104	96	113	918	198	186	211

Confidential: For Review Only

Supplementary Table 7 Time to PCR confirmed infection: hazard ratios (HR) by age group mutually adjusted for sex, socio-economic status, history of chronic conditions and parity and pre-term (age<5 years) and BMI (age 5-17 years)

	**Age <1 years**			**Age 1-4 years**			**Age 5-11 years**			**Age 12-17 years**		
N CYP in model	9396			47930			46753			61991		
N events in model												
	HR	95%LCI	95%UCI	HR	95%LCI	95%UCI	HR	95%LCI	95%UCI	HR	95%LCI	95%UCI
SEX												
Male	1.00	-	-	1.00	-	-	1.00	-	-	1.00	-	-
Female	1.14	0.92	1.40	1.09	0.97	1.21	1.09	1.00	1.19	1.21	1.14	1.28
SOCIO-ECONOMIC Position												
High	1.00	-	-	1.00	-	-	1.00	-	-	1.00	-	-
Middle	1.43	1.00	2.04	1.30	1.08	1.55	1.24	1.06	1.46	1.07	0.97	1.17
Low	1.46	1.01	2.10	1.34	1.11	1.61	1.15	0.98	1.34	0.95	0.87	1.05
CHRONIC CONDITIONS												
None	1.00	-	-	1.00	-	-	1.00	-	-	1.00	-	-
One	0.64	0.40	1.01	0.76	0.62	0.94	0.86	0.72	1.02	0.80	0.71	0.90
More than one	1.29	0.73	2.27	0.82	0.57	1.19	0.85	0.60	1.20	0.58	0.44	0.75
GESTATIONAL AGE												
pre-term	0.82	0.56	1.20	0.84	0.68	1.04	-	-	-	-	-	-
Term/post-erm	1.00	-	-	1.00	-	-	-	-	-	-	-	-
NUMBER OLDER SIBLINGS												
None	1.00	-	-	1.00	-	-	-	-	-	-	-	-
One	0.63	0.49	0.80	0.86	0.76	0.98	-	-	-	-	-	-
More than one	0.80	0.61	1.06	0.91	0.78	1.06	-	-	-	-	-	-
BMI												
underweight	-	-	-	-	-	-	1.03	0.72	1.48	1.04	0.84	1.28
normal	-	-	-	-	-	-	1.00	-	-	1.00	-	-
overweight/obese	-	-	-	-	-	-	1.06	0.96	1.18	1.08	1.01	1.15

Confidential: For Review Only

Supplementary Table 8 Rate of COVID-related admissions by age group per 100,000 CYP-years – extra variables

	**Age<1 years**				**Age 1-4 years**			
	Events	Rate	95%LCI	95%UCI	Events	Rate	95%LCI	95%UCI
	53	121	92	158	51	27	21	36
GESTATIONAL AGE								
pre-term	42	106	79	144	41	24	18	33
term/post-term	10	290	156	538	8	53	27	106
NUMBER OLDER SIBLINGS								
None	24	129	86	192	*	26	17	40
One	16	109	67	178	*	25	15	40
More than one	11	120	67	217	*	31	17	54
	**Age 5-11 years**				**Age 12-17 years**			
	Events	Rate	95%LCI	95%UCI	Events	Rate	95%LCI	95%UCI
	55	16	12	21	49	18	13	23
BMI								
underweight	*	54	13	215	*	24	*	168
normal	*	13	9	19	*	12	*	18
overweight/obese	*	15	8	29	*	25	14	44

*Redacted due to small numbers in some groups

Confidential: For Review Only

Supplementary Table 9 Time-to-COVID related admission: hazard ratios (HR) by age group mutually adjusted for sex, socio-economic status, history of chronic conditions and parity and pre-term (age<5 years) and BMI (aged 5-17 years)

	**Age <1 year**			**Age 1-4 years**			**Age 5-11 years**			**Age 12-17 years**		
N CYP in model	89197			244438			235396			316309		
N events in model												
	HR	95%LCI	95%UCI	HR	95%LCI	95%UCI	HR	95%LCI	95%UCI	HR	95%LCI	95%UCI
SEX												
Male	1.00	-	-	1.00	-	-	1.00	-	-	1.00	-	-
Female	0.81	0.50	1.31	1.58	0.92	2.71	0.71	0.34	1.50	0.87	0.50	1.52
SOCIO-ECONOMIC Position												
High	1.00	-	-	1.00	-	-	1.00	-	-	1.00	-	-
Middle	1.98	0.70	5.61	1.26	0.43	3.68	0.89	0.30	2.67	3.34	0.79	14.08
Low	2.46	0.87	6.95	1.91	0.67	5.45	0.52	0.16	1.67	2.49	0.59	10.62
CHRONIC CONDITIONS												
None	1.00	-	-	1.00	-	-	1.00	-	-	1.00	-	-
One	3.05	1.44	6.49	2.69	1.19	6.08	14.71	6.47	33.41	12.50	6.75	23.18
More than one	9.75	4.02	23.66	21.71	11.00	42.85	51.43	20.08	131.73	26.14	11.76	58.12
GESTATIONAL AGE												
pre-term	1.67	0.87	3.24	0.97	0.44	2.14	-	-	-	-	-	-
term	1.00	-	-	1.00	-	-	-	-	-	-	-	-
NUMBER OLDER SIBLINGS												
None	1.00	-	-	1.00	-	-	-	-	-	-	-	-
One	0.82	0.47	1.44	0.82	0.44	1.52	-	-	-	-	-	-
More than one	0.96	0.52	1.75	0.83	0.42	1.67	-	-	-	-	-	-
BMI												
underweight	-	-	-	-	-	-	1.75	0.23	13.05	2.22	0.53	9.27
normal	-	-	-	-	-	-	1.00	-	-	1.00	-	-
overweight/obese	-	-	-	-	-	-	0.94	0.40	2.21	1.41	0.76	2.60

Confidential: For Review Only

Supplementary Table 10 Rate of 'specific' admissions (age 0-4 years) per 100,000 CYP-years

	Age<1 years				Age 1-4 years			
	Events	Rate	95%LCI	95%UCI	Events	Rate	95%LCI	95%UCI
	47	107.0	80.4	142.4	25	13.4	9.0	19.8
SEX								
Male	26	115.4	78.6	169.5	10	10.4	5.6	19.3
Female	21	98.1	64.0	150.5	15	16.6	10.0	27.5
Socio-economic position								
High	*	72.8	27.3	193.9	*	9.0	2.3	36.0
Middle	*	104.0	68.5	157.9	*	12.5	6.9	22.5
Low	*	121.5	79.2	186.3	*	15.7	8.9	27.7
CHRONIC CONDITIONS								
None	*	98.2	72.3	133.4	*	11.7	7.6	18.1
One	*	221.8	83.3	591.0	*	23.1	7.4	71.5
More than one	*	516.0	129.0	2063.0	*	68.0	17.0	271.9
GESTATIONAL AGE								
pre-term	38	96.2	70.0	132.2	*	13.1	8.6	19.9
term/post-term	8	231.7	115.9	463.3	*	20.0	6.4	61.9
NUMBER OLDER SIBLINGS								
None	21	112.6	73.4	172.6	*	14.2	7.9	25.7
One	15	102.2	61.6	169.5	*	13.9	7.2	26.7
More than one	9	98.4	51.2	189.1	*	12.8	5.3	30.7
BMI								
underweight	-	-	-	-	-	-	-	-
normal	-	-	-	-	-	-	-	-
overweight/obese	-	-	-	-	-	-	-	-

*redacted due to small numbers in some groups

Confidential: For Review Only

Supplementary Table 11 Rate of 'specific' admissions (age 5-22 years) per 100,000 CYP-years

	**Age 5-11 years**				**Age 12-17 years**				**Age 18-22 years**			
	Events	Rate	95%LCI	95%UCI	Events	Rate	95%LCI	95%UCI	Events	Rate	95%LCI	95%UCI
	28	8.0	5.6	11.7	26	9.3	6.3	13.6	62	27.7	21.6	35.5
SEX												
Male	15	8.4	5.1	14.0	14	9.8	5.8	16.5	27	23.6	16.2	34.4
Female	13	7.7	4.4	13.2	12	8.8	5.0	15.5	35	31.9	22.9	44.4
Socio-economic position												
High	*	4.9	1.2	19.6	*	2.9	0.4	20.3	*	16.9	5.4	52.3
Middle	*	8.4	4.9	14.5	*	10.0	5.7	17.6	*	22.1	15.2	32.0
Low	*	8.5	5.0	14.7	*	10.4	6.0	17.9	*	39.0	27.4	55.5
CHRONIC CONDITIONS												
None	12	3.7	2.1	6.5	10	3.8	2.1	7.1	21	10.5	6.9	16.1
One	6	29.3	13.2	65.3	8	53.0	26.5	106.0	23	122.6	81.5	184.5
More than one	10	217.1	116.8	403.6	8	204.6	102.3	409.1	18	317.9	200.3	504.5
GESTATIONAL AGE												
pre-term	-	-	-	-	-	-	-	-	-	-	-	-
term	-	-	-	-	-	-	-	-	-	-	-	-
post-term	-	-	-	-	-	-	-	-	-	-	-	-
NUMBER OLDER SIBLINGS												
None	-	-	-	-	-	-	-	-	-	-	-	-
One	-	-	-	-	-	-	-	-	-	-	-	-
More than one	-	-	-	-	-	-	-	-	-	-	-	-
BMI												
underweight	*	53.9	13.5	215.4	*	0.0			-	-	-	-
normal	*	6.5	3.8	11.2	*	5.9	3.2	11.0	-	-	-	-
overweight/obese	*	10.0	4.5	22.3	*	12.4	5.6	27.7	-	-	-	-

*redacted due to small numbers in some groups

Confidential: For Review Only

Supplementary Table 12 Time-to-COVID related admission (specific definition): hazard ratios (HR) by age group mutually adjusted for sex, socio-economic status and history of chronic conditions (age 0-4)

	**Age <1 year**			**Age 1-4 years**		
N CYP in model	92530			251884		
N events in model	47			25		
	HR	95%LCI	95%UCI	HR	95%LCI	95%UCI
SEX						
Male	1.00	-	-	1.00	-	-
Female	0.77	0.46	1.31	1.89	0.87	4.14
SOCIO-ECONOMIC Position						
High	1.00	-	-	1.00	-	-
Middle	1.40	0.54	3.63	1.62	0.37	7.17
Low	1.54	0.59	4.03	1.69	0.38	7.57
CHRONIC CONDITIONS						
None	1.00	-	-	1.00	-	-
One	2.28	0.91	5.73	2.52	0.87	7.36
More than one	4.28	1.04	17.59	5.54	1.30	23.70

Confidential: For Review Only

Supplementary Table 13 Time-to-COVID related <u>admission (specific definition)</u>: hazard ratios (HR) by age group mutually adjusted for sex, socio-economic status and history of chronic conditions (age 5-22)

	**Age 5-11 years**			**Age 12-17 years**			**Age 18-22 years**		
N CYP in model	347542			385664			268467		
N events in model	28			26			62		
	HR	95%LCI	95%UCI	HR	95%LCI	95%UCI	HR	95%LCI	95%UCI
SEX									
Male	1.00	-	-	1.00	-	-	1.00	-	-
Female	1.26	0.56	2.82	0.98	0.51	1.86	1.07	0.64	1.79
SOCIO-ECONOMIC Position									
High	1.00	-	-	1.00	-	-	1.00	-	
Middle	1.36	0.30	6.07	4.58	0.61	34.46	1.02	0.31	3.37
Low	1.02	0.22	4.66	4.34	0.58	32.47	1.67	0.51	5.49
CHRONIC CONDITIONS									
None	1.00	-	-	1.00	-	-	1.00	-	
One	7.87	2.68	23.07	11.14	5.05	24.58	9.89	5.41	18.08
More than one	64.09	25.87	158.82	48.19	22.33	104.01	28.22	15.06	52.89

Confidential: For Review Only

Supplementary Figure 1 Flow chart describing creation of the final cohort

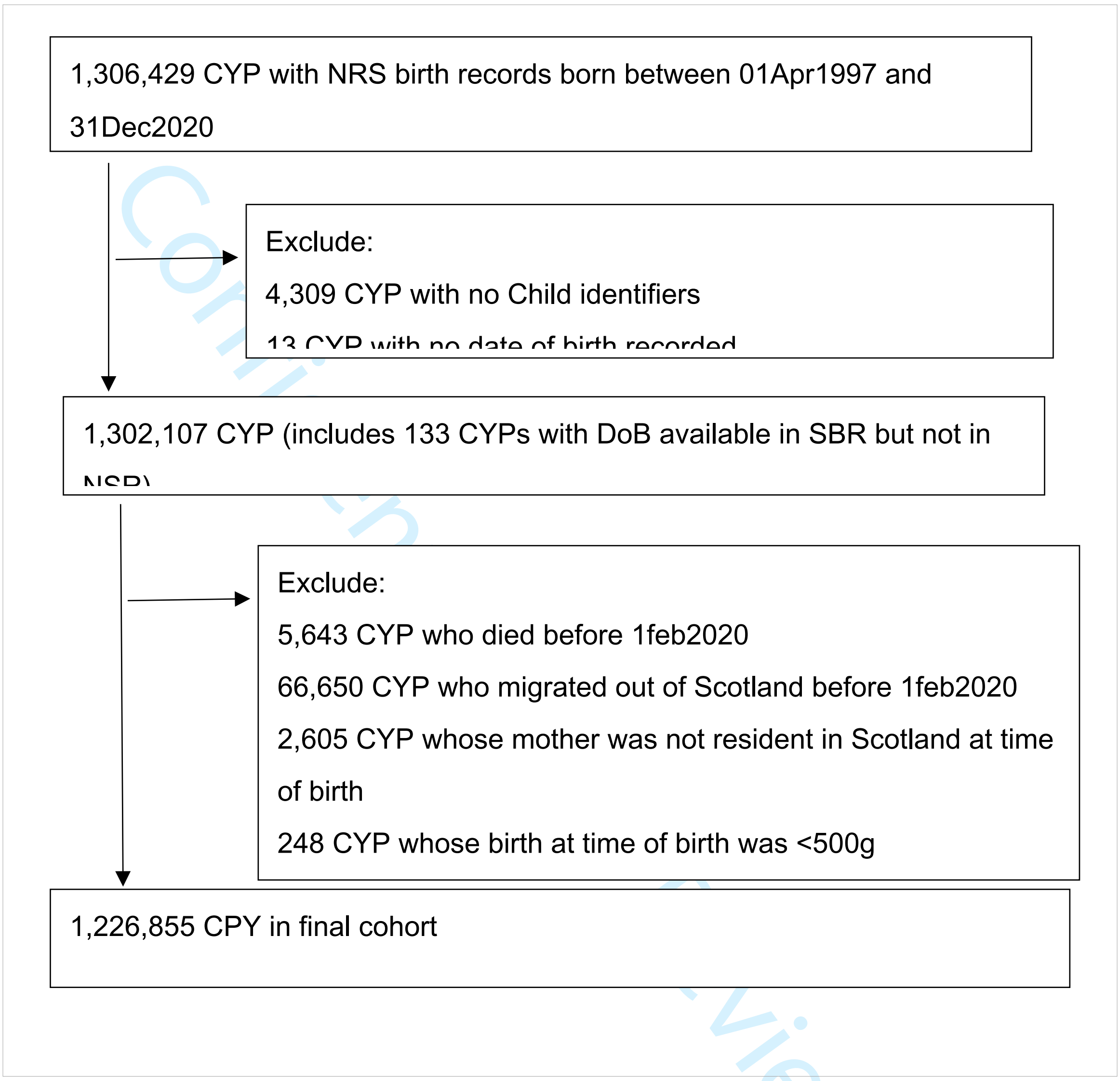

Supplementary Figure 2 Number of tests (bars) and positive tests (red) by age group and week of 2020

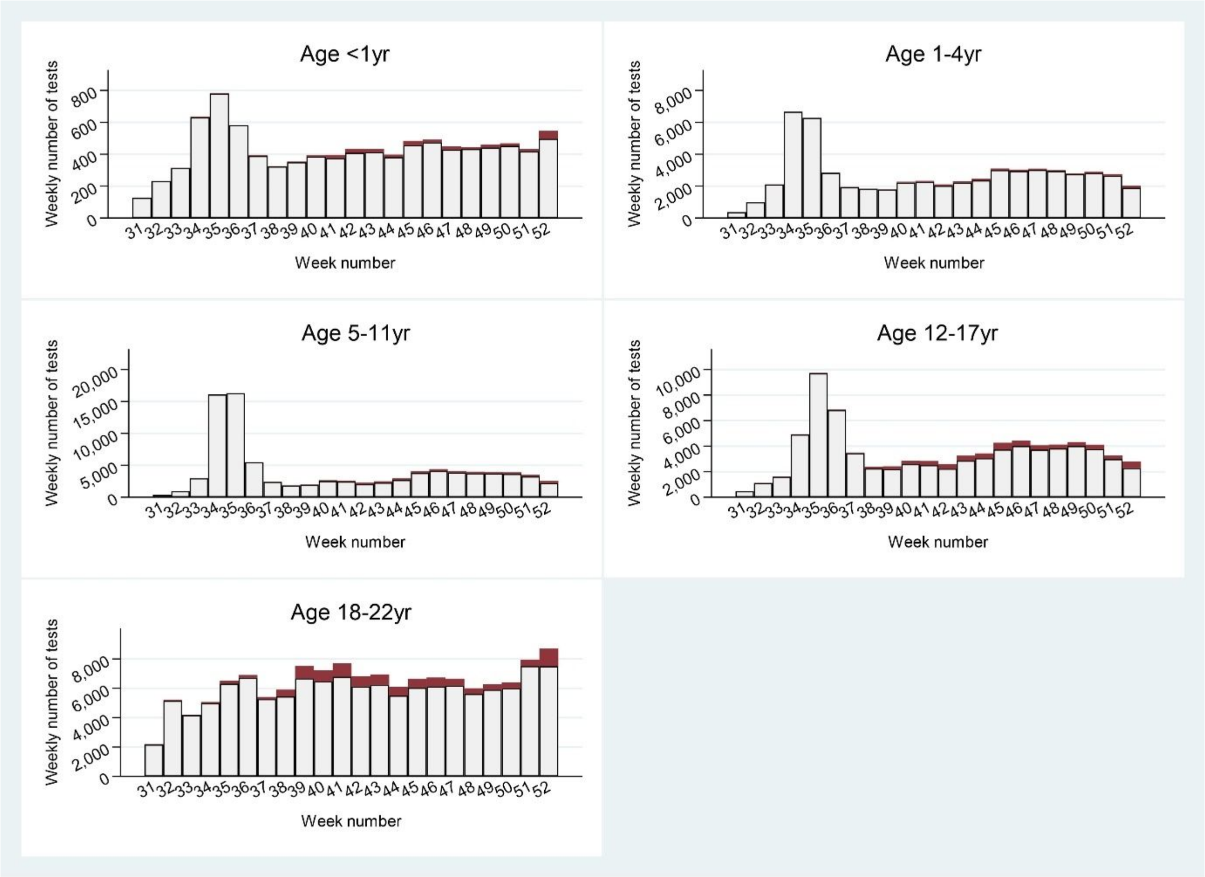

For Peer Review Only

BMJ Paediatrics Open

SARS-CoV-2 tests, confirmed infections and COVID-19 related hospital admissions in children and young people: birth cohort study

Journal:	*BMJ Paediatrics Open*
Manuscript ID	bmjpo-2022-001545.R1
Article Type:	Original research
Date Submitted by the Author:	08-Jul-2022
Complete List of Authors:	Hardelid, Pia; University College London Great Ormond Street Institute of Child Health, Population, Policy & Practice Research and Teaching Department<br>Favarato, Graziella; University College London Great Ormond Street Institute of Child Health, Population, Policy & Practice Research and Teaching Department<br>Wijlaars, Linda; University College London Great Ormond Street Institute of Child Health, Population, Policy & Practice Research and Teaching Department<br>Fenton, Lynda; Public Health Scotland, Clinical and Public Health Intelligence Team<br>McMenamin, Jim; Public Health Scotland, Respiratory Infection Team<br>Clemens, Tom; The University of Edinburgh, School of Geosciences<br>Dibben, Chris; The University of Edinburgh, School of Geosciences<br>Milojevic, Ai; London School of Hygiene & Tropical Medicine, Department of Public Health, Environments and Society<br>Macfarlane, Alison; City University of London<br>Taylor, Jonathon; Tampere University, Faculty of Built Environment<br>Cunningham, Steven; University of Edinburgh, Centre for Inflammation Research<br>Wood, Rachael; University of Edinburgh
Keywords:	COVID-19, Epidemiology

SCHOLARONE™
Manuscripts

I, the Submitting Author has the right to grant and does grant on behalf of all authors of the Work (as defined in the below author licence), an exclusive licence and/or a non-exclusive licence for contributions from authors who are: i) UK Crown employees; ii) where BMJ has agreed a CC-BY licence shall apply, and/or iii) in accordance with the terms applicable for US Federal Government officers or employees acting as part of their official duties; on a worldwide, perpetual, irrevocable, royalty-free basis to BMJ Publishing Group Ltd ("BMJ") its licensees and where the relevant Journal is co-owned by BMJ to the co-owners of the Journal, to publish the Work in this journal and any other BMJ products and to exploit all rights, as set out in our licence.

The Submitting Author accepts and understands that any supply made under these terms is made by BMJ to the Submitting Author unless you are acting as an employee on behalf of your employer or a postgraduate student of an affiliated institution which is paying any applicable article publishing charge ("APC") for Open Access articles. Where the Submitting Author wishes to make the Work available on an Open Access basis (and intends to pay the relevant APC), the terms of reuse of such Open Access shall be governed by a Creative Commons licence – details of these licences and which Creative Commons licence will apply to this Work are set out in our licence referred to above.

Other than as permitted in any relevant BMJ Author's Self Archiving Policies, I confirm this Work has not been accepted for publication elsewhere, is not being considered for publication elsewhere and does not duplicate material already published. I confirm all authors consent to publication of this Work and authorise the granting of this licence.

Confidential: For Review Only

SARS-CoV-2 tests, confirmed infections and COVID-19 related hospital admissions in children and young people: birth cohort study

Pia Hardelid,[1†] Graziella Favarato,[1] Linda Wijlaars,[1] Lynda Fenton,[2] Jim McMenamin,[3] Tom Clemens,[4] Chris Dibben, [4] Ai Milojevic,[5] Alison Macfarlane,[6] Jonathon Taylor,[7] Steven Cunningham,[8*] Rachael Wood.[9*]

[1]Population, Policy and Practice Research and Teaching Department, University College London Great Ormond Street Institute of Child Health, London, UK

[2]Clinical and Public Health Intelligence Team, Public Health Scotland, Edinburgh, UK

[3] Respiratory Infection Team, Public Health Scotland, Glasgow, UK

[4]School of Geosciences, The University of Edinburgh, Edinburgh, UK

[5]Department of Public Health, Environments and Society, London School of Hygiene & Tropical Medicine, London, UK

[6]Centre for Maternal and Child Health Research, City, University of London, London, UK

[7]Faculty of Built Environment, Tampere University, Tampere, Finland

[8]Centre for Inflammation Research, University of Edinburgh, Edinburgh, UK

[2,9]Centre for Clinical Brain Sciences, University of Edinburgh, Edinburgh, UK

**Joint last authors

[†]Corresponding author. Address for correspondence: [1]Population, Policy and Practice Research and Teaching Department, University College London Institute of Child Health, 30 Guilford Street, London WC1N 1EH, UK. p.hardelid@ucl.ac.uk

Confidential: For Review Only

Abstract

Background
There have been no population-based studies of SARS-CoV-2 testing, PCR-confirmed infections and COVID-19-related hospital admissions across the full paediatric age range. We examine the epidemiology of SARS-CoV-2 in children and young people (CYP) aged <23 years.

Methods
We used a birth cohort of all children born in Scotland since 1997, constructed via linkage between vital statistics, hospital records and SARS-CoV-2 surveillance data. We calculated risks of tests and PCR-confirmed infections per 1000 CYP-years between August and December 2020, and COVID-19-related hospital admissions per 100,000 CYP-years between February and December 2020. We used Poisson and Cox proportional hazards regression models to determine risk factors.

Results
Among the 1226855 CYP in the cohort, there were 378,402 tests (a rate of 770.8/1000 CYP years (95% confidence interval [768.4-773.3]), 19,005 PCR confirmed infections (179.4/1000 CYP years [176.9-182.0]) and 346 admissions (29.4/100,000CYP years [26.3-32.8]). Infants had the highest COVID-19-related admission rates. Chronic conditions, particularly multiple types of conditions, was strongly associated with COVID-19-related admissions across all ages. Overall, 49% of admitted CYP had at least one chronic condition recorded.

Conclusions
Infants, and CYP with chronic conditions are at highest risk of admission with COVID-19. Half of admitted CYP had chronic conditions. Studies examining COVID vaccine effectiveness among children with chronic conditions, and whether maternal vaccine during pregnancy prevents COVID-19 admissions in infants are urgently needed.

Confidential: For Review Only

What is already known on this topic

Children are less likely to suffer severe symptoms of SARS-CoV-2 infection than adults. There are few population-based studies of the epidemiology of SARS-CoV-2 in children not admitted to hospital.

What this study adds

Using a national birth cohort from Scotland during 2020, we found that children and young people with chronic conditions were more likely to be tested, but secondary school aged children with chronic conditions were less likely to have a confirmed infection. Infants and children/young people with chronic conditions were at highest risk of admission.

How this study might affect research, practice or policy

Studies examining COVID vaccine effectiveness among children with chronic conditions, and whether maternal vaccine during pregnancy prevents COVID-19 admissions in infants are urgently needed.

Confidential: For Review Only

Background

Children are much less likely to experience hospital admission and mortality related to severe acute respiratory syndrome coronavirus-2 (SARS-CoV-2) infection than adults.[1] In Europe in 2020, 1.7% of COVID-19-related hospital admissions were in children <19 years of age.[2] Over the course of the pandemic our understanding of how SARS-CoV-2 infection affects children has also improved. Children who experience more severe symptoms of SARS-CoV-2 may present with acute infection symptoms such as fever or cough[3-5] Other children may develop an acute inflammatory syndrome, Paediatric Inflammatory Syndrome Temporally associated with SARS-CoV-2 (PIMS-TS; also referred to as Multisystem Inflammatory Syndrome related to COVID; MIS-C), several weeks after initial infection.[6-8] Children aged <2 years old appear to be over-represented among children admitted to hospital with acute symptoms, whereas children aged 10 years or older account for the largest proportion of admitted PIMS-TS cases.[4 9]

Among children admitted to hospital with SARS-CoV-2 or PIMS-TS, those with specific chronic respiratory, neurological, gastrointestinal or cardiovascular conditions, and particularly children with multiple comorbidities, were at increased risk of Paediatric Intensive Care Unit (PICU) admission or death. Infants and teenagers appeared to have higher odds of these severe outcomes compared to children aged 1-4 years old.[10 11] A lower reported risk of severe disease and, until 2021, relatively lower rates of infection in children, have supported a narrative that the benefits and risks (primarily of myocarditis following second dose mRNA vaccines in young men[12 13]) of vaccinations in children are finely balanced.

Most studies of paediatric SARS-CoV-2 infection have been case series of infected or hospitalised children, making calculations of population-based risks of confirmed infections and associated admissions among different groups of children, including children with chronic conditions, impossible. Our aim was to provide population-based estimates of risk of SARS-CoV-2 testing, polymerase chain reaction (PCR)-confirmed infections and COVID-19 related admissions in children and young people (CYP) based on age, presence of chronic conditions, and socioeconomic status during 2020 that could support vaccination and other policy recommendations across the population of children and young people.

Methods

Data sources

We used a national birth cohort of all CYP born in Scotland from 1997 onwards, developed from administrative health datasets linked to public health surveillance data on SARS-CoV-2

Confidential: For Review Only

test results, originally constructed for the PICNIC study.[14] Birth registrations comprised the cohort spine, and CYP are linked over time and between databases using the Community Health Index number, a unique personal identifier recorded at all interactions with the Scottish National Health Service (NHS). Table 1 summarises the databases and variables used in this study.

Confidential: For Review Only

Table 1. Datasets and variables from the national Scottish birth cohort used in the study

Dataset	Dataset details	Variables used
National Records for Scotland (NRS) birth registrations	Vital registration data on all children born in Scotland and their parents, collected via registry offices	Week and year of birth; Baby Sex; Socio-economic position (parents' occupation at birth).
Scottish Morbidity Record (SMR)-01	Contains data on post-neonatal admissions and day cases to all NHS hospitals in Scotland	Admission and discharges dates; Primary and secondary diagnoses during admission; Type of hospital admission; Admission and discharge data from Intensive Care Unit (ICU).
SMR-02 (maternity records)	Contains data on all maternity admissions (including deliveries) in Scotland	Estimated gestational age; birth weight; number of older siblings (parity).
COVID-19 Tests	Contains data on all PCR- and antigen tests for SARS-Cov-2 with results and dates	Date of testing; Type of test; Result.
National Records for Scotland (NRS) death registrations	Vital registration data on children who died in Scotland	Date of death; Cause of death.
Scottish Birth Records (SBR)	Contains data on all children born in NHS hospitals, with data on neonatal admissions in and after April 2003	Diagnoses recorded at or shortly after birth ; Primary and secondary diagnoses at birth admission.
CHI register	Contains data on migration in/out Scotland	Migration outside Scotland.
Child Health Surveillance Programme-School	Contains data school health visits	Height and weight at age 5

Confidential: For Review Only

Study population and follow-up

We included CYP born in Scotland from 1st April 1997 to 31st December 2020. Children born at less than 24 weeks' gestation or with a birthweight <500 grammes were excluded,[15] as were CYP whose mothers were not resident in Scotland at the time of delivery, and CYP who migrated out of Scotland before 1st February 2020. For analyses of SARS-CoV-2 tests and positive test results (from now on referred to as PCR-confirmed infections), CYP were followed from birth or 1st August 2020 (whichever occurred last), until death, migration from Scotland, their 23rd birthday or 31st December 2020, whichever occurred first. 1st August 2020 was chosen as the follow-up start date for analyses of tests and PCR-confirmed infections since this is when testing for SARS-CoV-2 became commonly available in the community (rather than solely in hospitals) for children of all ages.[16] Children, like adults, were advised to seek PCR testing if they developed a continuous cough, high temperature or loss of sense of smell or taste.[17 18]For calculation and analyses of rates of COVID-19-related admissions, we used 1st February 2020 as the follow-up start date. This allowed us to include all COVID-19-related hospital admissions since the start of the pandemic.

Outcomes

Our primary outcomes were rates of SARS-CoV-2 PCR tests (positive or negative); PCR-confirmed SARS-CoV-2 infections; COVID19-related hospital admissions. Our secondary outcomes were PIMS-TS admissions and COVID19-related intensive care unit (ICU) stays. Supplementary Text 1 details how each of these outcomes were derived.

Risk factors

We examined four risk key factors for testing, confirmed infections and hospital admission outcomes: age group, sex, family socio-economic position and history of chronic conditions. Age as of 1st February 2020 was grouped into: <1 year (this also includes children born during 2020), 1-4 years, 5-11 years, 12-17 years and 18-22 years. We chose these age groups to reflect likely mixing patterns based on age (i.e. prior to formal childcare, nursery/preschool, primary school, secondary school, and higher/further education or work). Family socio-economic position was defined using parents' (father's, or mother's if the birth was not jointly registered) occupation recorded on birth registration, coded using the UK National Statistics Socio-economic Classification (NS-SEC).[19] We collapsed the NS-SEC classes into: high (managerial and professional occupations), middle (intermediate occupations) and low (routine and manual occupations) socio-economic position. We identified history of chronic conditions by examining International Classification of Disease

Confidential: For Review Only

version 10 (ICD-10) diagnostic codes recorded in the Scottish Morbidity Record (SMR-01) between 1 Jan 2015 and 31 January 2020. using an existing code list.[20] For children aged less than five years at the start February 2020 or born during 2020, we used all available SMR-01 data and any diagnoses recorded on Scottish Birth Records (SBR). Chronic conditions were classified into 8 types: developmental/mental health, blood/cancer, chronic infections, respiratory, metabolic/gastrointestinal/endocrine/genitourinary, musculoskeletal/skin neurological/sensory, and cardiac conditions. These were further grouped into none, one type of condition, and more than one type of chronic condition for analyses.

We further explored whether gestational age and the number of older siblings affected PCR confirmed infection and hospital admission risk in children aged <5 years, and Body Mass Index (BMI) in CYP aged 5-17. Gestational age was grouped as: preterm (<37 weeks) and term/late term (≥37 weeks). Number of older siblings (indicated by parity) was grouped as: no older siblings, one older sibling and two or more older siblings. BMI was derived from the Child Health Surveillance Programme -School dataset collected from children starting their first year at school (at age 5 years), and categorised[21] as underweight (<5th percentile), healthy weight (5th to <85th percentile) and overweight/obese (≥85th percentile).

Statistical analyses

We calculated rates of testing and PCR confirmed infections per 1,000 CYP years and hospital admission per 100,000 CYP-years with 95% confidence intervals stratified by each risk factor. We estimated the median length of stay with interquartile ranges (IQRs) for COVID-19 related hospital admissions. We calculated the proportion of children with a COVID-19 related who had a chronic condition recorded either at baseline, or during the COVID-19 admission.

We examined the association between risk factors and testing rates using Poisson regression models with robust standard errors to account for multiple tests per child. To examine the association between risk factors and PCR-confirmed SARS-CoV-2 infection, and COVID19-related admission risk we used Cox proportional hazards regression models. Where a child had multiple COVID-19-related admissions, only the first was included in the Cox proportional hazards models. For each primary outcome, we first fitted an overall model including all ages and age group, sex, socio-economic position and history of chronic conditions as risk factors. We tested for interaction with age group and each of the other main risk factors using the Wald test. Two-sided *p*-values <0.05 were considered statistically significant.

Confidential: For Review Only

We then fitted models for each primary outcome stratified by age group if a statistically significant interaction with age was identified for any of the other variables or if we identified non-proportional hazards. In further analyses for ages <5 years old we included parity and gestational age as additional risk factors in the models; and for ages 5-17 years we included BMI category. We tested the proportional hazards assumption of the Cox model by inspecting plots of Schoenfeld residuals[22] and survival curves according to each main risk factor.

As there was only a small number of events for our secondary outcomes, we report the number of cases, median length of stay and age (with IQRs) only. All analyses were based on complete cases, as only a small number of CYP were missing values for any of the main variables. All statistical analyses were performed using Stata 16.0.

Sensitivity analyses

We examined the number of COVID-19 hospital admissions that were identified as occurring up to 14 days after a positive SARS-CoV-2 test. We repeated the analyses for hospital admission risk using a more specific definition of a COVID-19-related admission restricted to emergency admissions with an ICD-10 code indicating COVID-19 (U07.1 or U07.2)[23] as the primary diagnosis.

Patient and public involvement

The PICNIC study has been presented to a number of parent groups, including the Great Ormond Street Hospital Biomedical Research Centre Parents and Carers Advisory Group, and a coffee morning for parents at Shelter's Birmingham Office. This COVID epidemiology substudy has not been specifically reviewed by parents.

Results

This study included 1,226,855 CYP (Supplementary Figure 1). The median age in February 2020 was 11 years (IQR 5-17), and 8.0% of the cohort (97,884/1,226,855 CYP) had at least one chronic condition recorded in their hospital or birth record in the previous five years (Supplementary Table 1).

SARS-CoV-2 testing

Between 1st August (week 31) to 31st December 2020 (week 52) we identified 378,402 PCR tests linked to 256,741 CYP; 20.9% of CYP in the cohort had at least one test. Supplementary Figure 2 shows the weekly number of PCR tests by age group. The crude

Confidential: For Review Only

testing rate was 770.8 (95%CI 768.4-773.3) per 1,000 CYP-years. The majority of CYP had been tested only once (200,288; 78.0%); 40,188 (15.7%) had been tested twice and 16,265 (6.3%) more than twice. Further results regarding rates of testing by week and risk factor can be found in Supplementary Text 2 and Supplementary Tables 2-5.

PCR-confirmed infections

Among the 378,402 PCR tests identified in the cohort, 20,003 (5.3%) were positive and 7,275 (1.9%) were void. Excluding multiple positive tests per CYP, this corresponds to 19,005 PCR confirmed index infections in 7.4% (19,005/ 256,741) of the CYP who were tested between 1st August 2020 and 31st December 2020.

The overall rate of PCR-confirmed infections was 179.4 (95% CI 176.9-182.0) per 1,000 CYP-years. Young adults (aged 18-22 years) had the highest rates of PCR-confirmed infections and those aged 1-4 years the lowest (Table 2). Infants had the highest PCR-confirmed infection rates among preschool children, otherwise infection rates were positively correlated with age. CYP with chronic conditions had a lower risk of PCR-confirmed infection, particularly among secondary school aged children (Supplementary Table 4 & Table 3). Age-group specific analyses showed that among preschool children, PCR-confirmed infection rates were higher among children from lower socio-economic backgrounds, whereas the opposite was observed among CYP aged 12 years and above (Table 3).

Confidential: For Review Only

Table 2. Rates of PCR confirmed infections (per 1,000 CYP-years) by age group, sex, socio-economic position, and history of chronic conditions

	Age<1 year			Age 1-4 years			Age 5-11 years			Age 12-17 years			Age 18-22 years		
	Events	Rate	95%CI	Events	Rate	95%CI	Events	Rate	95%CI	Events	Rate	95%CI	Events	Rate	95%CI
	223	80	70, 91	1136	62	58, 65	3039	96	93, 100	4929	193	188, 198	9687	350	344, 358
SEX															
Male	121	79	66, 94	595	60	55, 65	1545	91	87, 96	2313	179	172, 187	4487	365	355, 376
Female	102	81	67, 98	541	64	59, 69	1494	102	97, 108	2616	207	199, 215	5200	338	329, 348
SOCIO-ECONOMIC POSITION															
High	21	51	33, 78	141	51	43, 60	292	81	73, 91	503	175	161, 191	1181	481	455, 510
Middle	111	83	69, 100	571	63	58, 69	1446	102	97, 108	2231	202	194, 210	5510	353	344, 363
Low	91	88	71, 108	424	64	58, 70	1301	94	89, 100	2195	189	181, 197	2996	312	301, 324
CHRONIC CONDITIONS															
None	202	81	71, 94	1031	63	60, 67	2757	98	94, 102	4591	199	194, 205	8766	366	358, 373
One	10	44	24, 82	77	45	36, 57	225	87	76, 99	272	143	127, 161	735	267	249, 287
>One	11	130	72, 235	28	54	37, 78	57	74	57, 95	66	107	84, 136	186	202	175, 233

PCR, Polymerase Chain Reaction; CYP, Children and Young Person

Confidential: For Review Only

Table 3 Time to PCR confirmed infection: hazard ratios (HR) by age group mutually adjusted for sex, socio-economic position and history of chronic conditions

	Age <1 years		Age 1-4 years		Age 5-11 years		Age 12-17 years		Age 18-22 years	
Number of CYP in model	9661		49288		70245		76262		70212	
Number of PCR confirmed infections	276		1114		1286		2706		4338	
	AdjHR	95%CI	AdjHR	95%CI	AdjHR	95%CI	AdjHR	95%CI	AdjHR	95%CI
SEX										
Male	1	-	1	-	1	-	1	-	1	-
Female	1.15	0.93, 1.41	1.08	0.97, 1.20	1.10	1.02, 1.19	1.20	1.15, 1.27	0.96	0.92, 1.00
SOCIO-ECONOMIC POSITION										
High	1	-	1	-	1	-	1	-	1	-
Middle	1.44	1.02, 2.04	1.30	1.09, 1.55	1.26	1.10, 1.44	1.02	0.94, 1.11	0.75	0.70, 0.80
Low	1.46	1.02, 2.08	1.31	1.10, 1.58	1.17	1.02, 1.34	0.91	0.83, 0.98	0.66	0.62, 0.71
CHRONIC CONDITIONS										
None	1	-	1	-	1	-	1	-	1	-
One	0.62	0.39, 0.98	0.74	0.60, 0.91	0.87	0.75, 1.00	0.79	0.71, 0.88	0.76	0.70, 0.82
> One	1.23	0.72, 2.10	0.79	0.55, 1.12	0.76	0.58, 1.00	0.59	0.48, 0.73	0.58	0.50, 0.67

PCR, Polymerase Chain Reaction; Adj HR, Adjusted Hazard Ratio; CI, Confidence Intervals.

Confidential: For Review Only

Children aged <5 years with one older sibling had a reduced risk of a PCR confirmed infection compared to children with no older siblings (Supplementary Table 6 and 7). Further, in children aged 12-17 years, being overweight/obese increased the risk of a PCR-confirmed infection compared to being of normal BMI.

COVID19-related-hospital admissions

Between 1st February 2020 and 31 December 2020, there were 81,312 admissions in 55,940 CYP. 346 (0.6%) admissions in 318 CYP were identified as COVID-19-related. The median length of stay was 2 days (IQR 1-4 days). There were 110 admissions between February and July (31.8%) and 236 (68.2%) between August and December (Figure 1). 49.4% (*n*=157) of the 318 CYP admitted had at least one type of chronic condition recorded; and 23.3% (74 of 318; 46.5% of the 159 children with at least one chronic condition) had multiple types of chronic conditions recorded.

The overall COVID-19 related admission rate was 29.4/100,000 (95%CI 26.3-32.8) CYP years (Table 4). Infants had the highest COVID-19 related admission rate: 120.6/100,000 (95%CI 92.2-157.9). CYP with chronic conditions, and particularly children with more than one chronic condition type recorded, had the highest admission rates across all age groups.

Of the CYP with chronic conditions who had a COVID-19 related admission, neurological/sensory conditions were the common condition type recorded among children aged<12 years, whereas among those aged 12-22 years the most common conditions were developmental/mental health conditions.

Presence of one or more chronic condition significantly increased the risk of COVID-19-related admissions across all ages (Supplementary table 4). In age-stratified analyses, presence of a chronic condition remained the only statistically significant risk factor for COVID-19-related admission across all age groups (Table 5). We did not identify any statistically significant associations between prematurity, number of older siblings, or BMI category and COVID-19 related hospital admission risk (Supplementary Table 9); however the number of hospital admissions was low in this study.

Confidential: For Review Only

Table 4. Rates of COVID-related admissions (per 100,000CYP-years) by age group, sex, socio-economic position, and history of chronic conditions

	**Age<1 year**			**Age 1-4 years**			**Age 5-11 years**			**Age 12-17 years**			**Age 18-22 years**		
	Events	Rate	95%CI	Events	Rate	95%CI	Events	Rate	95%CI	Even ts	Rate	95%CI	Events	Rate	95%CI
	53	121	92, 158	51	27	21, 36	55	16	12, 21	49	18	13, 23	110	49	41, 67
SEX															
Male	30	133	93, 190	22	23	15, 35	31	17	12, 25	26	18	12, 27	43	38	28, 55
Female	23	107	71, 162	29	32	22, 46	24	14	9, 21	23	17	11, 25	67	61	48, 91
SOCIO-ECONOMIC POSITION															
High	*	73	27, 194	*	18	7, 48	*	12	5, 29	*	9	3, 27	6	34	15, 75
Middle	*	118	80, 175	*	24	16, 36	*	15	10, 22	*	18	12, 28	55	43	33, 69
Low	*	139	93, 207	*	34	23, 50	*	18	12, 26	*	19	13, 29	49	62	47, 86
CHRONIC CONDITIONS															
None	*	105	78,142	*	20	15, 29	21	7	4,10	19	7	5,11	42	21	16, 28
One	*	277	115,666	*	38	16, 92	19	93	59, 146	17	113	70, 181	37	197	143, 272
>One	*	1032	387,2749	*	374	207, 675	15	326	196, 540	13	332	193, 573	31	547	385, 778

PCR, Polymerase Chain Reaction; CYP, Children and Young Person

*Redacted due to small numbers in some groups

Confidential: For Review Only

Table 5 Time-to-COVID related admissions: hazard ratios (HR) by age group mutually adjusted for sex, socio-economic position and history of chronic conditions

	Age <1 year		Age 1-4 years		Age 5-11 years		Age 12-17 years		Age 18-22 years	
Number of CYP in model	92,530		251,884		347,542		385,664		268,467	
N Admissions	53		51		55		49		110	
	AdjHR	95%CI	AdjHR	95%CI	AdjHR	95%CI	AdjHR	95%CI	AdjHR	95%CI
SEX										
Male	1	-	1	-	1	-	1	-	1	-
Female	0.82	0.51, 1.30	1.49	0.88, 2.51	1.10	0.62, 1.96	1.05	0.67, 1.67	1.47	0.99, 2.17
Socio-economic position										
High	1	-	1	-	1	-	1	-	1	-
Middle	1.70	0.66, 4.36	1.39	0.48, 4.03	0.91	0.34, 2.41	3.05	0.94, 9.92	1.03	0.44, 2.39
Low	2.07	0.81, 5.28	1.97	0.69, 5.61	0.92	0.35, 2.43	2.75	0.85, 8.93	1.27	0.54, 2.99
CHRONIC CONDITIONS										
None	1	-	1	-	1	-	1	-	1	-
One	3.14	1.50, 6.58	2.46	1.10, 5.53	13.73	6.99, 26.96	13.85	8.12, 23.61	8.86	5.62, 13.96
More than one	10.99	4.74, 25.48	19.71	10.45, 37.19	49.81	24.30, 102.12	40.96	22.89, 73.30	25.39	15.80, 40.82

Adj HR, Adjusted Hazard Ratio; CI, Confidence Intervals.

Confidential: For Review Only

ICU admissions and PIMS-TS cases

Thirteen (3.8%) of the 346 COVID-related admissions involved an ICU attendance, accounting for 1.2% of the 1,238 ICU admissions in CYP during the study period. The vast majority of these admissions were in CYP with a history of one or more types of chronic conditions. The median age of CYP admitted to ICU was 14 years (IQR 9-19 years) and the median length of stay at ICU was 6 days (IQR 2-7 days).

We identified fewer than five admissions with a diagnosis suggestive of PIMS-TS and temporally associated with a positive PCR test (<28 days prior admission by definition), all in males with an age spanning from 9 to 14 years. The median length of stay at admission was 10 days (IQR 6-14).

Sensitivity analyses

Of the 346 COVID-19-related admissions 203 (58.7%) had a specific COVID-19 ICD-10 code as the primary diagnosis and 258 (74.6%) were temporally associated with a SARS-CoV-2 positive test (Figure 2). Using the more specific definition of a COVID-19 related-admission the admission rates were 107.0 (95% CI 80.4-142.4), 13.4 (9.0-19.8), 8.0 (5.6 – 11.7), 9.3 (6.3-13.6) and 27.7 (21.6-35.5) per 100,000 CYP years in age groups <1, 1-4, 5-11, 12-17 and 18-22 years respectively (Supplementary Table 10 &11). This was between 12% (in infants) and 50% (in children aged 5-11 years) lower than the more inclusive definition used in the main analyses. Presence of one or more chronic conditions remained the only significant risk factor for hospital admission with the specific definition (Supplementary Tables 12 & 13) across the age groups. The median length of stay remained 2 days (IQR 1-5).

Discussion

Over one fifth of CYP in Scotland had at least one SARS-CoV-2 PCR test during 2020, and 1.5% had a PCR-confirmed infection. CYP with chronic conditions were more likely to be tested, but secondary school aged CYP with chronic conditions were less likely to have a PCR confirmed infection. Whilst COVID-19 related hospital admissions were uncommon (less than 3 per 10,000 CYP admitted in 2020), infants and CYP with chronic conditions recorded had the highest COVID-19 related-admission rates.

The Scottish data linkage infrastructure allowed us to include data for all CYP born in Scotland since 1997, thereby minimising selection bias and loss to follow-up. We relied on linkage between hospital admission and public health surveillance data to define COVID-19-

Confidential: For Review Only

related admissions, allowing us to examine the robustness of our definitions in the linked data, rather than only relying on time difference between SARS-CoV-2 positive test and hospital admission alone. Indeed, we demonstrated that using a more specific definition of a COVID-related hospital admission (including only emergency hospital admissions with a specific COVID ICD10 code recorded as the primary diagnosis) decreased the rates by up to 50% compared to using a more sensitive definition based on either a recorded diagnosis or a positive SARS-CoV-2 PCR test up to 14 days before, or during, admission. We therefore recommend varying the definition of a COVID-related hospital admission via sensitivity analyses in studies using linked administrative data to ensure robustness of findings. By using a national birth cohort for this study, we examined variations in population-based rates of SARS-CoV-2 testing, PCR confirmed infections and COVID-19-related hospital admissions across the full CYP age range, rather than only examining risk factors for ICU admission and death in hospitalised children.[24]

This study included data from the first year of the pandemic, when wildtype (until November 2020), followed by Alpha (dominant from December 2020) SARS-CoV-2 variants were circulating in Scotland. Our aim was to examine risk factors for SARS-CoV-2 testing, PCR-positive tests and COVID-related hospital admissions during 2020; rather than according to variant given that the wildtype variant was circulating for the majority of the time period. This study will need to be repeated to examine the impact of later circulating variants, including Delta and Omicron, and changing transmission dynamics as vaccination of adults and re-opening of schools, nurseries and workplaces since 2021 appear to be concentrating virus circulation among younger age groups.[25] The UK roll out of COVID vaccination for children aged 12-15 years, started in July 2021,[26] and vaccination for all children aged 5-11 from February 2022,[27] is likely to change risks of hospital admission, particularly among children with chronic conditions. Further studies will need to examine whether the COVID-19 vaccination programme has amended the admission risks reported in this study. The results reported here provide baseline risks during the first pandemic year against which more recent data can be compared. Updates of our analyses are planned.

We based our classification of chronic conditions on coded information in SMR-01 and SBR records and may have missed some common conditions that are primarily managed in primary or community care settings, such as asthma and diabetes. Further, as we limited our lookback period to identify chronic conditions to 5 years, in order to avoid including conditions that may have resolved among older children, this may further have led to under ascertainment of chronic conditions. Despite the use of a national birth cohort, the number of children admitted to hospital with SARS-CoV-2 during 2020 was small, therefore we were

Confidential: For Review Only

unable to estimate admission risks in groups of children with specific chronic conditions. Our classification of socio-economic position was based on parental occupation derived from birth certificates, which may not reflect current socio-economic circumstances (e.g.in older CYP).

As this study was based on linked, routinely collected data from the Scottish SARS-CoV-2 surveillance programme, our analyses of PCR-positive results relied on CYP (or in the case of younger children, their parents) coming forward for testing. Testing was recommended in individuals with high temperature, continuous cough or a loss of taste or smell; children are less likely than adults to display these symptoms when infected with SARS-CoV-2.[28] Further, our results regarding PCR-confirmed infections need to be interpreted in the context of testing behaviour. We demonstrated that during 2020, testing was more common among higher socio-economic groups in preschool children, whereas in children aged 12 years and over, the lowest socio-economic groups were more likely to test. These differences in testing behaviour are likely explained by factors such as presence of infection, severity and duration of symptoms, accessibility of testing and implications of test results for work, school and childcare[29].

Infants had the highest admission risk. A systematic review has indicated that infants are also at highest risk of requiring PICU admission once in hospital with COVID-19 disease.[30] However, admission rates in infancy related to SARS-CoV-2 (1/1000 child-years) during 2020 were lower than admission rates associated with confirmed influenza (2/1000 child-years)[31] or respiratory syncytial virus infections (22/1000-child years).[32] Future research should examine how COVID-19 vaccination programmes for pregnant women and older children, and removal of non-pharmaceutical interventions to control population mixing, affect infant SARS-CoV-2 admission rates.

We demonstrated that a history of chronic conditions, and particularly living with multiple different types of chronic conditions, was the most prominent risk factor for COVID-19 related hospital admission rates among CYP. Further, CYP with chronic conditions were more likely to be tested than those without, but less likely to have a PCR confirmed infection. This may reflect lower threshold for testing among high-risk groups.

Pre- and primary school children from lower socio-economic backgrounds had higher risks of PCR-confirmed infection than children from higher socio-economic backgrounds. Younger children spend more time in the home with their parents, thus their risk of infection is therefore more strongly associated with their parents' occupation (and ability to work from home). In older CYP, we instead identified higher PCR-confirmed infection rates among

https://mc.manuscriptcentral.com/bmjpo

Confidential: For Review Only

children of higher socio-economic position, despite lower testing rates. This may be due to CYP from lower socio-economic position groups being less likely to attend post-16 education, including university. There were large outbreaks in universities in Scotland in the autumn of 2020, which led to a surge in case numbers in 18-22 year olds.[33] Linkage between SARS-CoV-2 test results, hospital admission and education data are required to confirm whether exposure in education settings can explain these differences in infection risk.

We did not find a statistically significant association between socio-economic position and risk of COVID-related admission. This is unlike some previous reports which have demonstrated higher all-age hospital admission rates in areas with higher area-level deprivation scores.[34] However, across all ages the vast majority of COVID-19 related admissions are in adults. As COVID-19 related admission rates in children are much lower than in adults, systematic differences in admission rates by socio-economic position among specific age groups of CYP are harder to detect, even when using national data. Further, as we used parental occupation to indicate socio-economic background, this may not reflect current socio-economic circumstances, as discussed above.

Our results showing that COVID-19 related admission rates in CYP peak in infancy indicates that further research and efforts to prevent COVID-19 admissions in children should include a focus on this age group. Pregnant women in Scotland are recommended to receive two doses of Pfizer/BioNTech COVID-19 vaccine. [35] As for pertussis[36 37] and influenza,[38 39] maternal vaccination during pregnancy could protect young babies from SARS-CoV-2 infection, however no studies to date have examined this. Further, given that CYP with chronic conditions are more likely to be admitted to hospital admission with COVID-19 disease than other CYP, studies monitoring the effectiveness of COVID-19 vaccines against severe outcomes in these high-risk groups are required to determine whether vaccination reduces the risk of admission.

We identified a peak in COVID-19-related hospital admissions in infants, and presence of chronic conditions as the strongest risk factor for hospital admissions in CYP, yet half of CYP admitted did not have any chronic conditions recorded. Further studies are urgently needed to examine whether maternal vaccine during pregnancy prevents COVID-19 admissions in infants. These data also provide baseline risks of infection and hospital admission for risk-benefit assessments of childhood vaccination, particularly for preschool children.

Confidential: For Review Only

Ethics statement

This study was approved by the University of Edinburgh School of Geosciences Ethics Committee (reference number 2020-401) and the Public Benefit and Privacy Panel for Health and Social Care (reference 1819-0049).

Funding:

This work was supported by UKRI-Medical Research Council, grant number MR/T016558/1

Acknowledgements

We are grateful to Professor Bianca De Stavola (UCL) for her advice on statistical modelling. The authors would like to acknowledge the support of the eDRIS Team (Public Health Scotland), and particularly Diane Rennie, for their involvement in obtaining approvals, provisioning and linking data and the use of the secure analytical platform within the National Safe Haven. This work uses data provided by patients and collected by the NHS as part of their care and support

Competing interests

None

Author contributions

PH conceived the study together with RW and SC. PH acquired the data supported by RW. GF and LW analysed the data. PH, GF and LW drafted the paper. All other authors read and commented on the paper. All authors have read and approved the final version.

Confidential: For Review Only

Figure legends

Figure 1 Monthly number of COVID-related hospital admissions (week 5-52, year 2020; 1st February 2020 to 31st December 2020)

Figure 2 Number of COVID-related admissions temporally associated with PCR positive test up to 14 days before admission and by primary and secondary COVID19 diagnosis (U07.1/U07.2) Total admissions (*n*)= 346

Confidential: For Review Only

References

1. Davies NG, Klepac P, Liu Y, et al. Age-dependent effects in the transmission and control of COVID-19 epidemics. *Nature Medicine* 2020;26(8):1205-11. doi: 10.1038/s41591-020-0962-9
2. European Centre for Disease Prevention and Control. COVID-19 in children and the role of school settings in transmission - first update. 2020 https://www.ecdc.europa.eu/sites/default/files/documents/COVID-19-in-children-and-the-role-of-school-settings-in-transmission-first-update_1.pdf Accessed: 02/03/2021
3. Swann OV, Holden KA, Turtle L, et al. Clinical characteristics of children and young people admitted to hospital with covid-19 in United Kingdom: prospective multicentre observational cohort study. *BMJ* 2020;370:m3249. doi: 10.1136/bmj.m3249
4. Götzinger F, Santiago-García B, Noguera-Julián A, et al. COVID-19 in children and adolescents in Europe: a multinational, multicentre cohort study. *The Lancet Child & Adolescent Health* 2020;4(9):653-61. doi: 10.1016/S2352-4642(20)30177-2
5. Irfan O, Muttalib F, Tang K, et al. Clinical characteristics, treatment and outcomes of paediatric COVID-19: a systematic review and meta-analysis. *Archives of Disease in Childhood* 2021;106(5):440-48. doi: 10.1136/archdischild-2020-321385
6. Toubiana J, Levy C, Allali S, et al. Association between SARS-CoV-2 infection and Kawasaki-like multisystem inflammatory syndrome: a retrospective matched case–control study, Paris, France, April to May 2020. *Eurosurveillance* 2020;25(48):2001813. doi: doi:https://doi.org/10.2807/1560-7917.ES.2020.25.48.2001813
7. Belot A, Antona D, Renolleau S, et al. SARS-CoV-2-related paediatric inflammatory multisystem syndrome, an epidemiological study, France, 1 March to 17 May 2020. *Eurosurveillance* 2020;25(22):2001010. doi: doi:https://doi.org/10.2807/1560-7917.ES.2020.25.22.2001010
8. Whittaker E, Bamford A, Kenny J, et al. Clinical Characteristics of 58 Children With a Pediatric Inflammatory Multisystem Syndrome Temporally Associated With SARS-CoV-2. *JAMA* 2020;324(3):259-69. doi: 10.1001/jama.2020.10369
9. Flood J, Shingleton J, Bennett E, et al. Paediatric multisystem inflammatory syndrome temporally associated with SARS-CoV-2 (PIMS-TS): Prospective, national surveillance, United Kingdom and Ireland, 2020. *The Lancet Regional Health – Europe* 2021;3 doi: 10.1016/j.lanepe.2021.100075
10. Harwood R, Yan H, Da Camara NT, et al. Which children and young people are at higher risk of severe disease and death after SARS-CoV-2 infection: a systematic review and individual patient meta-analysis. *medRxiv* 2021:2021.06.30.21259763. doi: 10.1101/2021.06.30.21259763
11. Ward JL, Harwood R, Smith C, et al. Risk factors for intensive care admission and death amongst children and young people admitted to hospital with COVID-19 and PIMS-TS in England during the first pandemic year. *medRxiv* 2021:2021.07.01.21259785. doi: 10.1101/2021.07.01.21259785
12. Witberg G, Barda N, Hoss S, et al. Myocarditis after Covid-19 Vaccination in a Large Health Care Organization. *New England Journal of Medicine* 2021;385(23):2132-39. doi: 10.1056/NEJMoa2110737
13. Simone A, Herald J, Chen A, et al. Acute Myocarditis Following COVID-19 mRNA Vaccination in Adults Aged 18 Years or Older. *JAMA Internal Medicine* 2021;181(12):1668-70. doi: 10.1001/jamainternmed.2021.5511
14. Favarato G, Clemens T, Cunningham S, et al. Air Pollution, housing and respiratory tract Infections in Children: NatIonal birth Cohort study (PICNIC): study protocol. *BMJ Open* 2021;11(5):e048038. doi: 10.1136/bmjopen-2020-048038
15. Hardelid P, Verfuerden M, McMenamin J, et al. The contribution of child, family and health service factors to respiratory syncytial virus (RSV) hospital admissions in the first 3 years of life: birth cohort study in Scotland, 2009 to 2015. *Eurosurveillance*

Confidential: For Review Only

2019;24(1):1800046. doi: doi:https://doi.org/10.2807/1560-7917.ES.2019.24.1.1800046
16. Fenton L, Gribben C, Caldwell D, et al. Risk of hospital admission with covid-19 among teachers compared with healthcare workers and other adults of working age in Scotland, March 2020 to July 2021: population based case-control study. *BMJ* 2021;374:n2060. doi: 10.1136/bmj.n2060
17. Scotland PH. Expansion of COVID-19 testing. 2020 https://www.gov.scot/news/expansion-of-covid-19-testing/ Accessed: 08/07/2022
18. Public Health Scotland. COVID-19 testing for under-fives. 2020 https://www.gov.scot/news/covid-19-testing-for-under-fives/ Accessed: 08/07/2022
19. Office for National Statistics. The National Statistics Socio-economic classification (NS-SEC); 2010 https://www.ons.gov.uk/methodology/classificationsandstandards/otherclassifications/thenationalstatisticssocioeconomicclassificationnssecrebasedonsoc2010 Accessed 08/07/202220. Hardelid P, Davey J, Dattani N, et al. Child deaths due to injury in the four UK countries: a time trends study from 1980 to 2010. *PLoS One* 2013;8(7):e68323. doi: 10.1371/journal.pone.0068323
21. Cole TJ, Freeman JV, Preece MA. British 1990 growth reference centiles for weight, height, body mass index and head circumference fitted by maximum penalized likelihood. *Stat Med* 1998;17(4):407-29.
22. SCHOENFELD D. Partial residuals for the proportional hazards regression model. *Biometrika* 1982;69(1):239-41. doi: 10.1093/biomet/69.1.239
23. Public Health Scotland. Scottish Clinical Coding Standards – ICD-10. 2020 https://www.isdscotland.org/Products-and-Services/Terminology-Services/Clinical-Coding-Guidelines/Docs/SCCS-24-April-2020.pdf Accessed:
24. Griffith GJ, Morris TT, Tudball MJ, et al. Collider bias undermines our understanding of COVID-19 disease risk and severity. *Nature Communications* 2020;11(1):5749. doi: 10.1038/s41467-020-19478-2
25. Public Health Scotland. COVID-19 Daily Dashboard; 2021 https://public.tableau.com/app/profile/phs.covid.19/viz/COVID-19DailyDashboard_15960160643010/Overview ; Accessed 08/07/2022

26. Joint Committee of Vaccination and Immunisation. JCVI statement on COVID-19 vaccination of children and young people aged 12 to 17 years: 15 July 2021; 2021 https://www.gov.uk/government/publications/covid-19-vaccination-of-children-and-young-people-aged-12-to-17-years-jcvi-statement/jvci-statement-on-covid-19-vaccination-of-children-and-young-people-aged-12-to-17-years-15-july-2021 Accessed 08/07/2022
27. Joint Committee on Vaccination and Immunisation. JCVI statement on vaccination of children aged 5 to 11 years old. 2022 https://www.gov.uk/government/publications/jcvi-update-on-advice-for-covid-19-vaccination-of-children-aged-5-to-11/jcvi-statement-on-vaccination-of-children-aged-5-to-11-years-old Accessed: 21/03/2022
28. Fragaszy E, Shrotri M, Geismar C, et al. Symptom profiles and accuracy of clinical case definitions for COVID-19 in a community cohort: results from the Virus Watch study [version 1; peer review: awaiting peer review]. *Wellcome Open Research* 2022;7(84) doi: 10.12688/wellcomeopenres.17479.1
29. Smith LE, Potts HWW, Amlôt R, et al. Adherence to the test, trace, and isolate system in the UK: results from 37 nationally representative surveys. *BMJ* 2021;372:n608. doi: 10.1136/bmj.n608
30. Harwood R, Yan H, Talawila Da Camara N, et al. Which children and young people are at higher risk of severe disease and death after hospitalisation with SARS-CoV-2 infection in children and young people: A systematic review and individual patient meta-analysis. *eClinicalMedicine* 2022;44 doi: 10.1016/j.eclinm.2022.101287

31. Hardelid P, Verfuerden M, McMenamin J, et al. Risk factors for admission to hospital with laboratory-confirmed influenza in young children: birth cohort study. *Eur Resp J* 2017;50(3) doi: 10.1183/13993003.00489-2017
32. Hardelid P, Verfuerden M, McMenamin J, et al. The contribution of child, family and health service factors to respiratory syncytial virus (RSV) hospital admissions in the first 3 years of life: birth cohort study in Scotland, 2009 to 2015. *Euro Surveill* 2019;24(1) doi: 10.2807/1560-7917.ES.2019.24.1.1800046
33. Public Health Scotland. COVID-19 in Scotland Daily Dashboard: Trends and demographics; 2021 https://public.tableau.com/app/profile/phs.covid.19/viz/COVID-19DailyDashboard_15960160643010/Overview Accessed 08/007/2022
34. The Scottish Parliament. Health Inequality and COVID-19 in Scotland. 2021 https://digitalpublications.parliament.scot/ResearchBriefings/Report/2021/3/23/ee202c60-93ad-4a27-a6e7-67613856ba24 Accessed: 27/10/2021
35. UK Health Security Agency. Green Book Chapter 14a: COVID-192021.
36. Dabrera G, Amirthalingam G, Andrews N, et al. A Case-Control Study to Estimate the Effectiveness of Maternal Pertussis Vaccination in Protecting Newborn Infants in England and Wales, 2012–2013. *Clinical Infectious Diseases* 2014;60(3):333-37. doi: 10.1093/cid/ciu821
37. Sandmann F, Jit M, Andrews N, et al. Infant Hospitalizations and Fatalities Averted by the Maternal Pertussis Vaccination Program in England, 2012–2017: Post-implementation Economic Evaluation. *Clinical Infectious Diseases* 2020;71(8):1984-87. doi: 10.1093/cid/ciaa165
38. Benowitz I, Esposito DB, Gracey KD, et al. Influenza Vaccine Given to Pregnant Women Reduces Hospitalization Due to Influenza in Their Infants. *Clinical Infectious Diseases* 2010;51(12):1355-61. doi: 10.1086/657309
39. Mølgaard-Nielsen D, Fischer TK, Krause TG, et al. Effectiveness of maternal immunization with trivalent inactivated influenza vaccine in pregnant women and their infants. *Journal of Internal Medicine* 2019;286(4):469-80. doi: https://doi.org/10.1111/joim.12947

Confidential: For Review Only

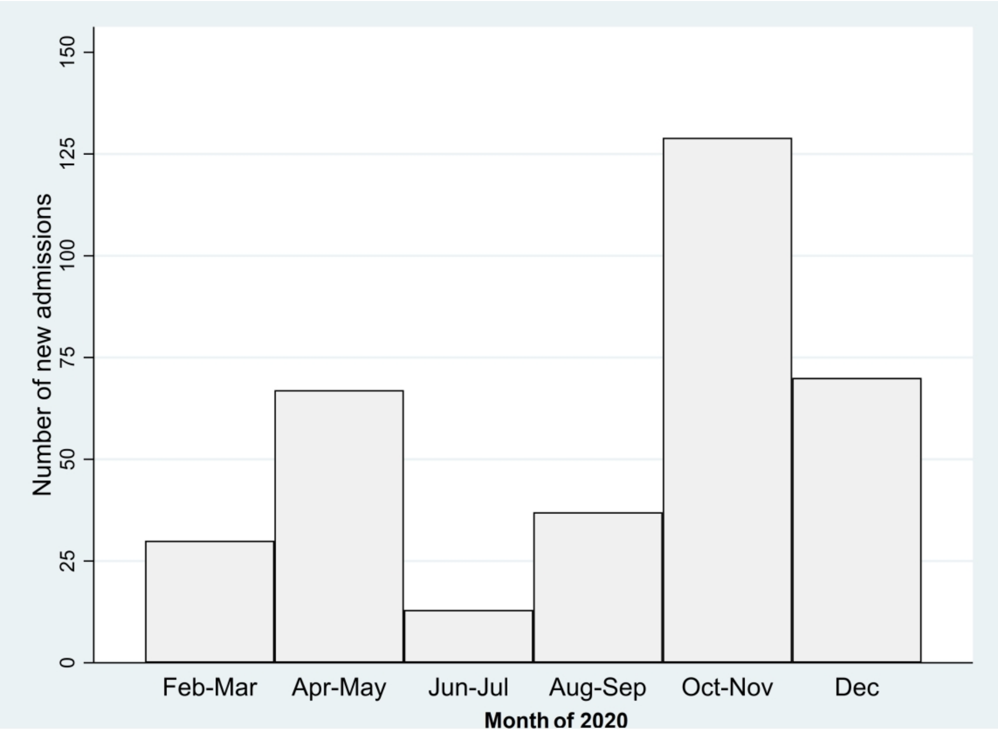

387x282mm (216 x 216 DPI)

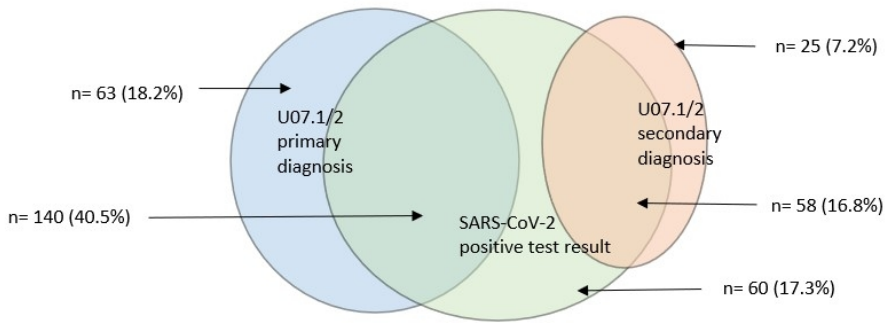

153x59mm (144 x 144 DPI)

SARS-CoV-2 tests, confirmed infections and hospital admissions in children and young people: birth cohort study

Supplementary material

Supplementary text 1

Outcome definitions

We included all SARS-CoV-2 PCR tests recorded between1st August 2020 and 31st December 2020 in the COVID19 Tests Dataset. The samples were collected in hospitals, primary care, via national testing centres, or self-collection via home test kits. We did not include antigen (lateral flow device) test results, as only 5% of test results in the cohort during the study period were from antigen tests. We defined as duplicate tests multiple tests taken on the same day, in the same CYP, with the same result, irrespective of whether they were taken at different locations. All duplicate tests, whether positive or negative, were excluded when calculating testing rates. A PCR confirmed infection was defined as the first record of a positive SARS-CoV-2 PCR test result (the index positive test) recorded in the COVID19 Tests dataset between1st August 2020 and 31st December 2020. Public Health Scotland recommends excluding all repeat positive tests within 90 days of the index positive sample date, and less than 5 CYP had multiple positive results beyond this time period. Therefore, only the first positive SARS-CoV-2 PCR test result for each child was included when calculating rates of PCR-confirmed SARS-CoV-2 infections.

We included all COVID-19-related hospital admissions between 1st February and 31st December 2020. To define COVID-19 related hospital admissions, we first linked episodes in the hospital admission dataset (Scottish Morbidity Record-01; Table 1) into admissions by assuming that episodes where the difference between the admission date and previous discharge date was ≤1 day[22] indicated the same admission. Second, we identified COVID-19 related admissions where: (i) an individual had tested positive for SARS-CoV-2 up to 14 days prior to hospital admission, on the day of admission, or in between the hospital admission and discharge date, and/or (ii) an International Classification of Diseases-version 10 (ICD-10) diagnostic code for COVID-19 (U07.1 – U07.2) had been recorded during an admission as a primary or secondary diagnosis.

https://mc.manuscriptcentral.com/bmjpo

Confidential: For Review Only

Since the ICD-10 code for PIMS-TS (U07.5) was introduced at the end of the follow-up period, we used other ICD-10 codes indicating systemic inflammatory response syndrome of infectious origin without organ failure (R65X), cardiogenic shock (R57X) or other specified systemic involvement of connective tissue (M35.8), suggestive of PIMS-TS recorded during an admission which had a positive SARS-CoV-2 PCR test within 28 day prior to the admission date.

A COVID-19-related intensive care unit (ICU) stay was defined where a child had an SMR-01 episode with ‘significant facility’ recorded with a positive SARS-CoV-2 PCR test to 21 days prior to the start of, or during, the ICU stay. ICU episodes where the difference between the ICU admission date and previous ICU discharge date was ≤1 day were assumed to indicate the same ICU stay.

Confidential: For Review Only

Supplementary text 2

Risk factors for testing

Testing rates varied by age group and chronic conditions; it was higher in children aged 1-4 years, young adults (age 18-22 years), and those with more than one chronic condition (Supplementary Table 2 & 3). Among children aged <5 years old, testing rates were higher in children from a higher socio-economic position, whereas among CYP aged 12-22 years, testing rates were higher in lower socio-economic groups.

The all-age model suggested increasing age and chronic conditions were strongly associated with being tested (Supplementary Table 4). In age-group stratified analyses, a history of chronic conditions was strongly associated with higher testing rates (Supplementary Table 5), particularly among infants.

Confidential: For Review Only

Supplementary Tables

Supplementary TABLE 1: Cohort baseline characteristics (*n*=1,226,855)

	Number	%
Sex		
Male	628,410	51.2
Female	598,445	48.8
missing	*0*	*0*
Age (years)*		
Median 10.8 years (IQR 5-17) y		
<1 year	92,539	7.5
1-4 years	206,677	16.9
5-11 years	326,455	26.6
12-17 years	358,195	29.2
18-22 years	242,989	19.8
Missing	*0*	*0*
Socio economic position**		
High	136,938	11.2
Middle	582,342	47.5
Low	507,563	41.4
Missing	*12*	*0*
Chronic conditions***		
None	1,128,971	92.0
One	78,016	6.4
More than one type	19,868	1.6
Gestational age (weeks) (aged* <5yr, *n*=292,289)		
Pre-term (<37 weeks)	23,825	8.0
Normal/Post-term (≥37 weeks)	268,464	89.7
Missing	*6,927*	*2.3*
Number of older siblings (aged*<5yr, *n*=289,800)		
None	124,289	41.5
One	102,944	34.4
Two or more	62,567	20.9
Missing	*9,416*	3.2
BMI *** (aged* 5-17, *n*=550,874)		
Underweight	8,930	1.30
Normal	421,182	60.2
Overweight/Obese	120,762	17.6
missing	*142,776*	*20.9*

* As on 1st February 2020; aged<1yr includes those born between 1 Feburary2020 and 31 December 2020. ** From UK National Statistics Socio-economic Classification (NS-SEC): SEP (managerial and professional occupations), middle SEP (intermediate occupations), low SEP (routine and manual occupations). ***Includes any chronic conditions recorded in the hospital records in the previous five years. ***As recorded in the Child Health Surveillance Programme-School at aged 5 and standardised according to the British 1990 growth reference standards (Cole 1998): underweight (<5th percentile), normal weight (5th to <85th percentile), overweight/obese (≥85th percentile).

Confidential: For Review Only

Supplementary Table 2 Rate testing by age group (age 0-4 years) per 1,000 CYP-years

	**Age <1 year**				**Age 1-4 years**			
	Events	Rate	95%LCI	95%UCI	Events	Rate	95%LCI	95%UCI
Overall	9509	482	473	492	59176	702	696	708
SEX								
Male	5256	519	506	534	32284	743	735	752
Female	4253	443	430	457	26892	658	650	666
Socio-economic position								
High	1368	551	522	581	9417	937	918	956
Middle	4417	466	452	480	29119	729	720	737
Low	3724	481	466	497	20637	602	594	610
CHRONIC CONDITIONS								
None	7678	408	399	417	50790	657	651	663
One	980	1323	1242	1408	5712	997	971	1023
More than one	851	5481	5124	5861	2674	2082	2005	2163
GESTATIONAL AGE								
pre-term	8099	457	448	468	52447	693	687	699
Term/post-term	1207	797	753	843	5566	818	796	839
NUMBER OLDER SIBLINGS								
None	4030	479	464	494	26920	770	761	779
One	3182	489	472	506	20177	692	682	701
More than one	1996	487	466	509	10371	589	577	600
BMI								
underweight	-	-	-	-	-	-	-	-
normal	-	-	-	-	-	-	-	-
overweight/obese	-	-	-	-	-	-	-	-

Confidential: For Review Only

Supplementary Table 3 Rate of testing by age group (age 5-22 years) per 1,000 CYP-years

	**Age 5-11 years**				**Age 12-17 years**				**Age 18-22 years**			
	Events	Rate	95%LCI	95%UCI	Events	Rate	95%LCI	95%UCI	Events	Rate	95%LCI	95%UCI
Overall	92007	583	580	587	79771	623	619	627	137939	1364	1357	1371
SEX												
Male	49757	622	616	628	45322	586	581	592	46290	895	887	903
Female	42250	558	553	564	46831	635	630	641	91649	1854	1842	1866
Socio-economic position												
High	10528	591	579	603	10059	531	521	541	9961	1198	1175	1222
Middle	41274	593	587	600	39678	612	606	618	78068	1390	1380	1399
Low	40203	588	582	595	42416	631	625	637	49909	1362	1350	1374
CHRONIC CONDITIONS												
None	80747	552	549	556	70707	592	587	596	117695	1303	1296	1311
One	8000	850	832	869	6395	944	921	967	14918	1789	1761	1818
More than one	3260	1538	1486	1592	2669	1530	1473	1590	5326	2137	2080	2195
GESTATIONAL AGE												
pre-term	-	-	-	-	-	-	-	-	-	-	-	-
term	-	-	-	-	-	-	-	-	-	-	-	-
post-term	-	-	-	-	-	-	-	-	-	-	-	-
NUMBER OLDER SIBLINGS												
None	-	-	-	-	-	-	-	-	-	-	-	-
One	-	-	-	-	-	-	-	-	-	-	-	-
More than one	-	-	-	-	-	-	-	-	-	-	-	-
BMI												
underweight	1009	626	589	666	1250	644	610	681	-	-	-	-
normal	49281	567	562	572	46792	594	589	600	-	-	-	-
overweight/obese	15287	588	579	598	14415	639	628	649	-	-	-	-

Confidential: For Review Only

Supplementary Table 4 Results of models adjusted for age group, sex, socio-economic position, and history of chronic conditions

	Testing*		PCR confirmed infection**		Admission***	
	Adj IRR	95%CI	Adj HR	95%CI	Adj HR	95%CI
AGE GROUP						
<1year	0.94	0.92, 0.95	0.76	0.70, 0.84	10.11	7.14, 14.32
1-4 years	1.14	1.12, 1.15	0.58	0.54, 0.62	1.11	0.74, 1.68
5-11 years	1.00	-	1	-	1	-
12-17 years	1.15	1.13, 1.16	2.38	2.28, 2.48	1.25	0.87, 1.80
18-22 years	1.89	1.87, 1.91	3.13	3.00, 3.26	2.32	1.66, 3.23
SEX						
Male	1	-	1	-	1	-
Female	1.16	1.15, 1.17	1.05	1.02, 1.08	1.11	0.89, 1.39
Socio-economic position						
High	1	-	1	-	1	-
Middle	1.00	0.98, 1.01	0.95	0.91, 1.00	1.34	0.86, 2.11
Low	0.96	0.95, 0.98	0.85	0.81 0.89	1.53	0.98. 2.40
CHRONIC CONDITIONS						
None	1	-	1	-	1	-
One	1.38	1.36, 1.40	0.75	0.71, 0.79	7.55	5.79, 9.86
More than one	1.85	1.81, 1.88	0.61	0.55, 0.68	26.17	19.79, 34.59

PCR, Polymerase Chain Reaction; Adj IRR, Adjusted Incidence Risk Ratio; Adj HR, Adjusted Hazard Ratio; CI, Confidence Intervals.

Footnotes:

* Wald test for interaction: age and sex p<0.0001; age and socio-economic position p<0.0001; age and chronic conditions p<0.0001;

**interaction: age and sex p<0.0001 , age and socio-economic position p<0.0001 , age and chronic conditions p=0.0004; global test to check proportionality assumption p<0.0001 (for age, socio-economic position, chronic conditions).

*** interaction: age and sex p= 0.045 , age and NS-SEC p= 0.94 , age and chronic conditions p =0.26; global test to check proportionality assumption p 0.0009 (for age, chronic condition

Confidential: For Review Only

Supplementary Table 5 Incidence Risk Ratio of being tested by age group mutually adjusted for sex, socio-economic status, history of chronic conditions and parity and pre-term (age<5 years) and BMI (aged 5-17 years)

	**Age <1 year**			**Age 1-4 years**			**Age 5-11 years**			**Age 12-17 years**		
N CYP in model	89202			200590			310670			231202		
N tests in model	94799			213258			323020			247143		
	IRR	95%LCI	95%UCI	IRR	95%LCI	95%UCI	IRR	95%LCI	95%UCI	IRR	95%LCI	95%UCI
SEX												
Male	1.00	-	-	1.00	-	-	1.00	-	-	1.00	-	-
Female	0.89	0.86	0.92	0.91	0.90	0.93	0.91	0.89	0.92	1.15	1.13	1.17
SOCIO-ECONOMIC POSITION												
High	1.00	-	-	1.00	-	-	1.00	-	-	1.00	-	-
Middle	0.76	0.73	0.80	0.84	0.82	0.86	1.02	1.00	1.05	1.09	1.06	1.13
Low	0.62	0.60	0.65	0.76	0.74	0.78	1.01	0.98	1.03	1.10	1.06	1.13
CHRONIC CONDITIONS												
None	1.00	-	-	1.00	-	-	1.00	-	-	1.00	-	-
One	2.13	2.02	2.25	1.44	1.40	1.48	1.46	1.42	1.49	1.48	1.43	1.53
More than one	3.89	3.66	4.14	2.22	2.11	2.33	2.14	2.03	2.25	1.91	1.80	2.02
GESTATIONAL AGE												
Pre-term	1.10	1.04	1.15	1.07	1.04	1.10	-	-	-	-	-	-
Term/post-term	1.00	-	-	1.00	-	-	-	-	-	-	-	-
NUMBER OLDER SIBLINGS												
None	1.00	-	-	1.00	-	-	-	-	-	-	-	-
One	0.99	0.95	1.02	0.91	0.89	0.93	-	-	-	-	-	-
More than one	0.86	0.82	0.90	0.82	0.80	0.84	-	-	-	-	-	-
BMI												
underweight	-	-	-	-	-	-	1.04	0.98	1.11	1.10	1.02	1.19
normal	-	-	-	-	-	-	1.00	-	-	1.00	-	-
overweight/obese	-	-	-	-	-	-	1.03	1.01	1.04	1.05	1.03	1.08

Confidential: For Review Only

Supplementary Table 6 Rate of PCR confirmed infections by age group per 1,000 CYP-years – extra variables

	**Age<1 year**				**Age 1-4 years**			
	Events	Rate	95%LCI	95%UCI	Events	Rate	95%LCI	95%UCI
	223	80	70	91	1136	62	58	65
GESTATIONAL AGE								
pre-term	192	78	68	90	1022	62	59	66
term/post-term	24	85	57	126	89	53	43	66
NUMBER OLDER SIBLINGS								
None	109	92	76	110	541	65	60	71
One	58	60	46	77	358	57	51	63
More than one	48	86	65	114	201	61	53	70
	**Age 5-11 years**				**Age 12-17 years**			
	Events	Rate	95%LCI	95%UCI	Events	Rate	95%LCI	95%UCI
	3039	96	93	100	4929	193	188	198
BMI								
underweight	38	112	82	154	72	179	142	225
normal	1723	101	96	106	2791	182	175	189
overweight/obese	545	104	96	113	918	198	186	211

Confidential: For Review Only

Supplementary Table 7 Time to PCR confirmed infection: hazard ratios (HR) by age group mutually adjusted for sex, socio-economic status, history of chronic conditions and parity and pre-term (age<5 years) and BMI (age 5-17 years)

	**Age <1 years**			**Age 1-4 years**			**Age 5-11 years**			**Age 12-17 years**		
N CYP in model	9396			47930			46753			61991		
N events in model												
	HR	95%LCI	95%UCI	HR	95%LCI	95%UCI	HR	95%LCI	95%UCI	HR	95%LCI	95%UCI
SEX												
Male	1.00	-	-	1.00	-	-	1.00	-	-	1.00	-	-
Female	1.14	0.92	1.40	1.09	0.97	1.21	1.09	1.00	1.19	1.21	1.14	1.28
SOCIO-ECONOMIC Position												
High	1.00	-	-	1.00	-	-	1.00	-	-	1.00	-	-
Middle	1.43	1.00	2.04	1.30	1.08	1.55	1.24	1.06	1.46	1.07	0.97	1.17
Low	1.46	1.01	2.10	1.34	1.11	1.61	1.15	0.98	1.34	0.95	0.87	1.05
CHRONIC CONDITIONS												
None	1.00	-	-	1.00	-	-	1.00	-	-	1.00	-	-
One	0.64	0.40	1.01	0.76	0.62	0.94	0.86	0.72	1.02	0.80	0.71	0.90
More than one	1.29	0.73	2.27	0.82	0.57	1.19	0.85	0.60	1.20	0.58	0.44	0.75
GESTATIONAL AGE												
pre-term	0.82	0.56	1.20	0.84	0.68	1.04	-	-	-	-	-	-
Term/post-erm	1.00	-	-	1.00	-	-	-	-	-	-	-	-
NUMBER OLDER SIBLINGS												
None	1.00	-	-	1.00	-	-	-	-	-	-	-	-
One	0.63	0.49	0.80	0.86	0.76	0.98	-	-	-	-	-	-
More than one	0.80	0.61	1.06	0.91	0.78	1.06	-	-	-	-	-	-
BMI												
underweight	-	-	-	-	-	-	1.03	0.72	1.48	1.04	0.84	1.28
normal	-	-	-	-	-	-	1.00	-	-	1.00	-	-
overweight/obese	-	-	-	-	-	-	1.06	0.96	1.18	1.08	1.01	1.15

Confidential: For Review Only

Supplementary Table 8 Rate of COVID-related admissions by age group per 100,000 CYP-years – extra variables

	**Age<1 years**				**Age 1-4 years**			
	Events	Rate	95%LCI	95%UCI	Events	Rate	95%LCI	95%UCI
	53	121	92	158	51	27	21	36
GESTATIONAL AGE								
pre-term	42	106	79	144	41	24	18	33
term/post-term	10	290	156	538	8	53	27	106
NUMBER OLDER SIBLINGS								
None	24	129	86	192	*	26	17	40
One	16	109	67	178	*	25	15	40
More than one	11	120	67	217	*	31	17	54
	**Age 5-11 years**				**Age 12-17 years**			
	Events	Rate	95%LCI	95%UCI	Events	Rate	95%LCI	95%UCI
	55	16	12	21	49	18	13	23
BMI								
underweight	*	54	13	215	*	24	*	168
normal	*	13	9	19	*	12	*	18
overweight/obese	*	15	8	29	*	25	14	44

*Redacted due to small numbers in some groups

Confidential: For Review Only

BMJ Paediatrics Open

Supplementary Table 9 Time-to-COVID related admission: hazard ratios (HR) by age group mutually adjusted for sex, socio-economic status, history of chronic conditions and parity and pre-term (age<5 years) and BMI (aged 5-17 years)

	**Age <1 year**			**Age 1-4 years**			**Age 5-11 years**			**Age 12-17 years**		
N CYP in model	89197			244438			235396			316309		
N events in model												
	HR	95%LCI	95%UCI	HR	95%LCI	95%UCI	HR	95%LCI	95%UCI	HR	95%LCI	95%UCI
SEX												
Male	1.00	-	-	1.00	-	-	1.00	-	-	1.00	-	-
Female	0.81	0.50	1.31	1.58	0.92	2.71	0.71	0.34	1.50	0.87	0.50	1.52
SOCIO-ECONOMIC Position												
High	1.00	-	-	1.00	-	-	1.00	-	-	1.00	-	-
Middle	1.98	0.70	5.61	1.26	0.43	3.68	0.89	0.30	2.67	3.34	0.79	14.08
Low	2.46	0.87	6.95	1.91	0.67	5.45	0.52	0.16	1.67	2.49	0.59	10.62
CHRONIC CONDITIONS												
None	1.00	-	-	1.00	-	-	1.00	-	-	1.00	-	-
One	3.05	1.44	6.49	2.69	1.19	6.08	14.71	6.47	33.41	12.50	6.75	23.18
More than one	9.75	4.02	23.66	21.71	11.00	42.85	51.43	20.08	131.73	26.14	11.76	58.12
GESTATIONAL AGE												
pre-term	1.67	0.87	3.24	0.97	0.44	2.14	-	-	-	-	-	-
term	1.00	-	-	1.00	-	-	-	-	-	-	-	-
NUMBER OLDER SIBLINGS												
None	1.00	-	-	1.00	-	-	-	-	-	-	-	-
One	0.82	0.47	1.44	0.82	0.44	1.52	-	-	-	-	-	-
More than one	0.96	0.52	1.75	0.83	0.42	1.67	-	-	-	-	-	-
BMI												
underweight	-	-	-	-	-	-	1.75	0.23	13.05	2.22	0.53	9.27
normal	-	-	-	-	-	-	1.00	-	-	1.00	-	-
overweight/obese	-	-	-	-	-	-	0.94	0.40	2.21	1.41	0.76	2.60

Confidential: For Review Only

Supplementary Table 10 Rate of 'specific' admissions (age 0-4 years) per 100,000 CYP-years

	Age<1 years				Age 1-4 years			
	Events	Rate	95%LCI	95%UCI	Events	Rate	95%LCI	95%UCI
	47	107.0	80.4	142.4	25	13.4	9.0	19.8
SEX								
Male	26	115.4	78.6	169.5	10	10.4	5.6	19.3
Female	21	98.1	64.0	150.5	15	16.6	10.0	27.5
Socio-economic position								
High	*	72.8	27.3	193.9	*	9.0	2.3	36.0
Middle	*	104.0	68.5	157.9	*	12.5	6.9	22.5
Low	*	121.5	79.2	186.3	*	15.7	8.9	27.7
CHRONIC CONDITIONS								
None	*	98.2	72.3	133.4	*	11.7	7.6	18.1
One	*	221.8	83.3	591.0	*	23.1	7.4	71.5
More than one	*	516.0	129.0	2063.0	*	68.0	17.0	271.9
GESTATIONAL AGE								
pre-term	38	96.2	70.0	132.2	*	13.1	8.6	19.9
term/post-term	8	231.7	115.9	463.3	*	20.0	6.4	61.9
NUMBER OLDER SIBLINGS								
None	21	112.6	73.4	172.6	*	14.2	7.9	25.7
One	15	102.2	61.6	169.5	*	13.9	7.2	26.7
More than one	9	98.4	51.2	189.1	*	12.8	5.3	30.7
BMI								
underweight	-	-	-	-	-	-	-	-
normal	-	-	-	-	-	-	-	-
overweight/obese	-	-	-	-	-	-	-	-

*redacted due to small numbers in some groups

Confidential: For Review Only

Supplementary Table 11 Rate of 'specific' admissions (age 5-22 years) per 100,000 CYP-years

	**Age 5-11 years**				**Age 12-17 years**				**Age 18-22 years**			
	Events	Rate	95%LCI	95%UCI	Events	Rate	95%LCI	95%UCI	Events	Rate	95%LCI	95%UCI
	28	8.0	5.6	11.7	26	9.3	6.3	13.6	62	27.7	21.6	35.5
SEX												
Male	15	8.4	5.1	14.0	14	9.8	5.8	16.5	27	23.6	16.2	34.4
Female	13	7.7	4.4	13.2	12	8.8	5.0	15.5	35	31.9	22.9	44.4
Socio-economic position												
High	*	4.9	1.2	19.6	*	2.9	0.4	20.3	*	16.9	5.4	52.3
Middle	*	8.4	4.9	14.5	*	10.0	5.7	17.6	*	22.1	15.2	32.0
Low	*	8.5	5.0	14.7	*	10.4	6.0	17.9	*	39.0	27.4	55.5
CHRONIC CONDITIONS												
None	12	3.7	2.1	6.5	10	3.8	2.1	7.1	21	10.5	6.9	16.1
One	6	29.3	13.2	65.3	8	53.0	26.5	106.0	23	122.6	81.5	184.5
More than one	10	217.1	116.8	403.6	8	204.6	102.3	409.1	18	317.9	200.3	504.5
GESTATIONAL AGE												
pre-term	-	-	-	-	-	-	-	-	-	-	-	-
term	-	-	-	-	-	-	-	-	-	-	-	-
post-term	-	-	-	-	-	-	-	-	-	-	-	-
NUMBER OLDER SIBLINGS												
None	-	-	-	-	-	-	-	-	-	-	-	-
One	-	-	-	-	-	-	-	-	-	-	-	-
More than one	-	-	-	-	-	-	-	-	-	-	-	-
BMI												
underweight	*	53.9	13.5	215.4	*	0.0			-	-	-	-
normal	*	6.5	3.8	11.2	*	5.9	3.2	11.0	-	-	-	-
overweight/obese	*	10.0	4.5	22.3	*	12.4	5.6	27.7	-	-	-	-

*redacted due to small numbers in some groups

Confidential: For Review Only

Supplementary Table 12 Time-to-COVID related <u>admission (specific definition)</u>: hazard ratios (HR) by age group mutually adjusted for sex, socio-economic status and history of chronic conditions (age 0-4)

	**Age <1 year**			**Age 1-4 years**		
N CYP in model	92530			251884		
N events in model	47			25		
	HR	95%LCI	95%UCI	HR	95%LCI	95%UCI
SEX						
Male	1.00	-	-	1.00	-	-
Female	0.77	0.46	1.31	1.89	0.87	4.14
SOCIO-ECONOMIC Position						
High	1.00	-	-	1.00	-	-
Middle	1.40	0.54	3.63	1.62	0.37	7.17
Low	1.54	0.59	4.03	1.69	0.38	7.57
CHRONIC CONDITIONS						
None	1.00	-	-	1.00	-	-
One	2.28	0.91	5.73	2.52	0.87	7.36
More than one	4.28	1.04	17.59	5.54	1.30	23.70

Confidential: For Review Only

Supplementary Table 13 Time-to-COVID related admission (specific definition): hazard ratios (HR) by age group mutually adjusted for sex, socio-economic status and history of chronic conditions (age 5-22)

	Age 5-11 years			Age 12-17 years			Age 18-22 years		
N CYP in model	347542			385664			268467		
N events in model	28			26			62		
	HR	95%LCI	95%UCI	HR	95%LCI	95%UCI	HR	95%LCI	95%UCI
SEX									
Male	1.00	-	-	1.00	-	-	1.00	-	-
Female	1.26	0.56	2.82	0.98	0.51	1.86	1.07	0.64	1.79
SOCIO-ECONOMIC Position									
High	1.00	-	-	1.00	-	-	1.00	-	
Middle	1.36	0.30	6.07	4.58	0.61	34.46	1.02	0.31	3.37
Low	1.02	0.22	4.66	4.34	0.58	32.47	1.67	0.51	5.49
CHRONIC CONDITIONS									
None	1.00	-	-	1.00	-	-	1.00	-	
One	7.87	2.68	23.07	11.14	5.05	24.58	9.89	5.41	18.08
More than one	64.09	25.87	158.82	48.19	22.33	104.01	28.22	15.06	52.89

Confidential: For Review Only

Supplementary Figure 1 Flow chart describing creation of the final cohort

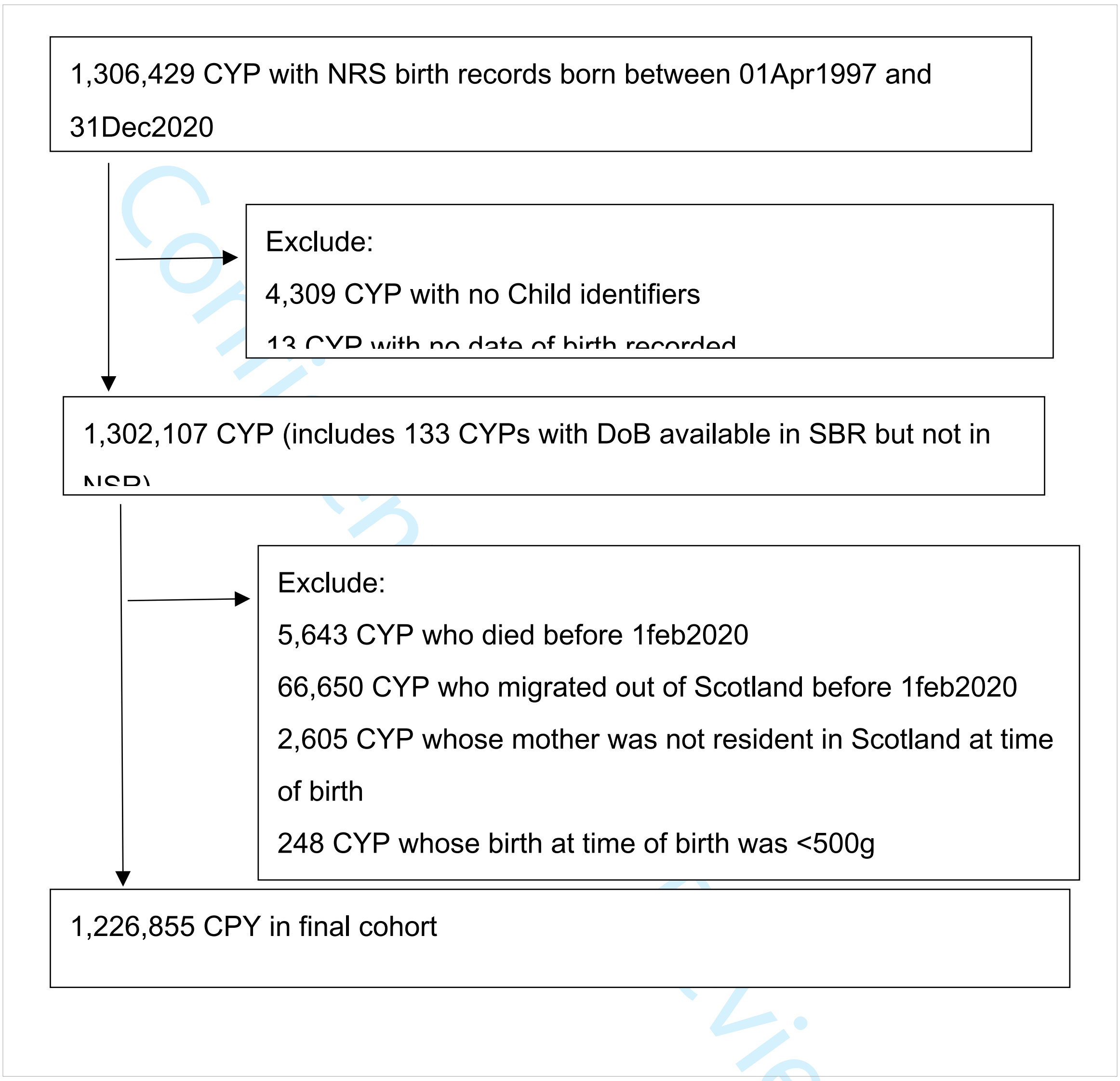

For peer review only

Supplementary Figure 2 Number of tests (bars) and positive tests (red) by age group and week of 2020 (note that the scale of the y-axis is not the same for all graphs)

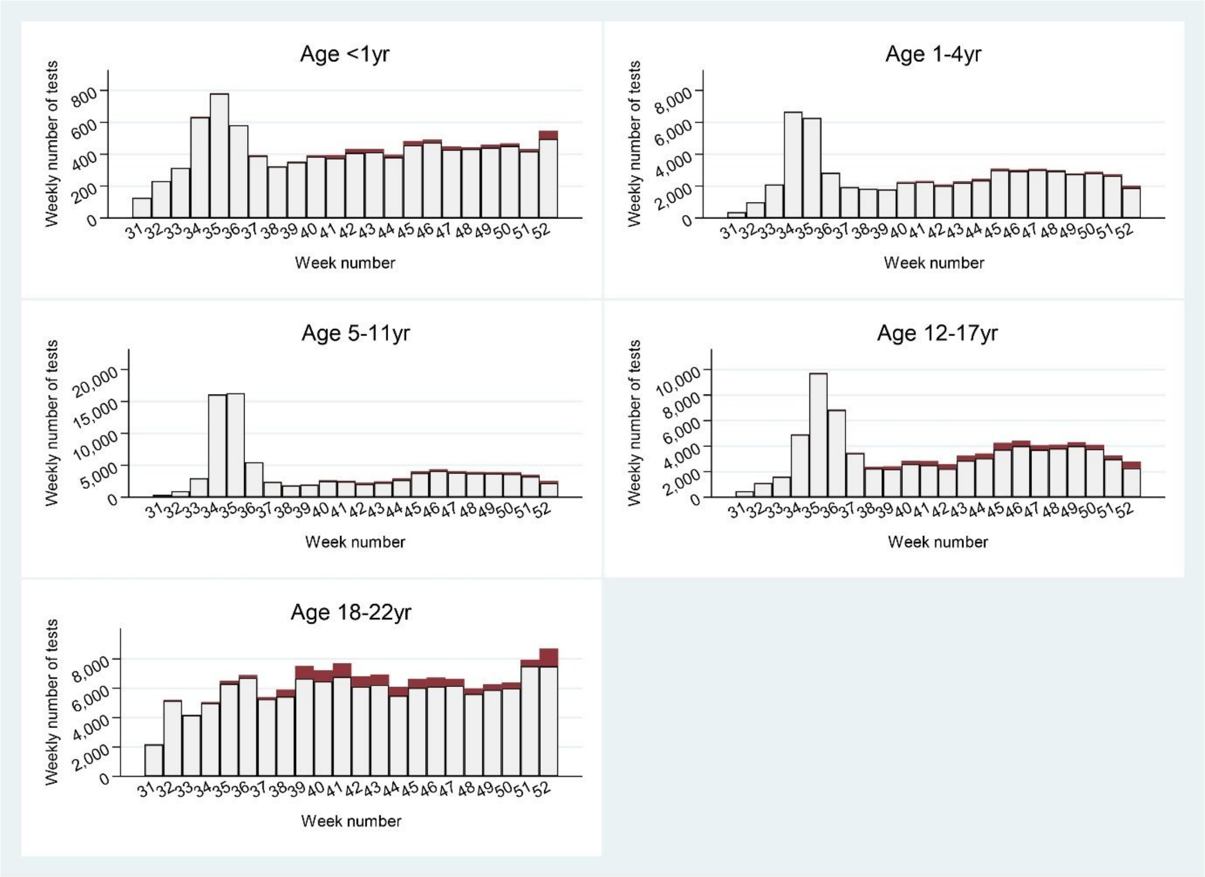

For Peer Review Only
